# Design principles for site-selective hydroxylation by a Rieske oxygenase

**DOI:** 10.1038/s41467-021-27822-3

**Published:** 2022-01-11

**Authors:** Jianxin Liu, Jiayi Tian, Christopher Perry, April L. Lukowski, Tzanko I. Doukov, Alison R. H. Narayan, Jennifer Bridwell-Rabb

**Affiliations:** 1 Department of Chemistry, University of Michigan, Ann Arbor, MI 48109 USA; 2 Program in Chemical Biology, University of Michigan, Ann Arbor, MI 48109 USA; 3 Life Sciences Institute, University of Michigan, Ann Arbor, MI 48109 USA; 4 Macromolecular Crystallography Group, Stanford Synchrotron Radiation Light Source, SLAC National Accelerator Laboratory, Menlo Park, CA 94025 USA; 5 Present Address: Center for Marine Biotechnology and Biomedicine, Scripps Institution of Oceanography, University of California, San Diego, La Jolla, CA 92037 USA

**Keywords:** X-ray crystallography, Enzyme mechanisms, Iron, Protein design

## Abstract

Rieske oxygenases exploit the reactivity of iron to perform chemically challenging C–H bond functionalization reactions. Thus far, only a handful of Rieske oxygenases have been structurally characterized and remarkably little information exists regarding how these enzymes use a common architecture and set of metallocenters to facilitate a diverse range of reactions. Herein, we detail how two Rieske oxygenases SxtT and GxtA use different protein regions to influence the site-selectivity of their catalyzed monohydroxylation reactions. We present high resolution crystal structures of SxtT and GxtA with the native β-saxitoxinol and saxitoxin substrates bound in addition to a Xenon-pressurized structure of GxtA that reveals the location of a substrate access tunnel to the active site. Ultimately, this structural information allowed for the identification of six residues distributed between three regions of SxtT that together control the selectivity of the C–H hydroxylation event. Substitution of these residues produces a SxtT variant that is fully adapted to exhibit the non-native site-selectivity and substrate scope of GxtA. Importantly, we also found that these selectivity regions are conserved in other structurally characterized Rieske oxygenases, providing a framework for predictively repurposing and manipulating Rieske oxygenases as biocatalysts.

## Abstract (web summary)

SxtT and GxtA are Rieske oxygenases that are involved in paralytic shellfish toxin biosynthesis and catalyze monohydroxylation reactions at different positions on the toxin scaffold. Here, the authors present crystal structures of SxtT and GxtA with the native substrates β-saxitoxinol and saxitoxin as well as a Xenon-pressurized structure of GxtA, which reveal a substrate access tunnel to the active site. Through structure-based mutagenesis studies the authors identify six residues in three different protein regions that determine the substrate specificity and site selectivity of SxtT and GxtA. These findings will aid the rational engineering of other Rieske oxygenases.

## Introduction

**Fig. 1:**
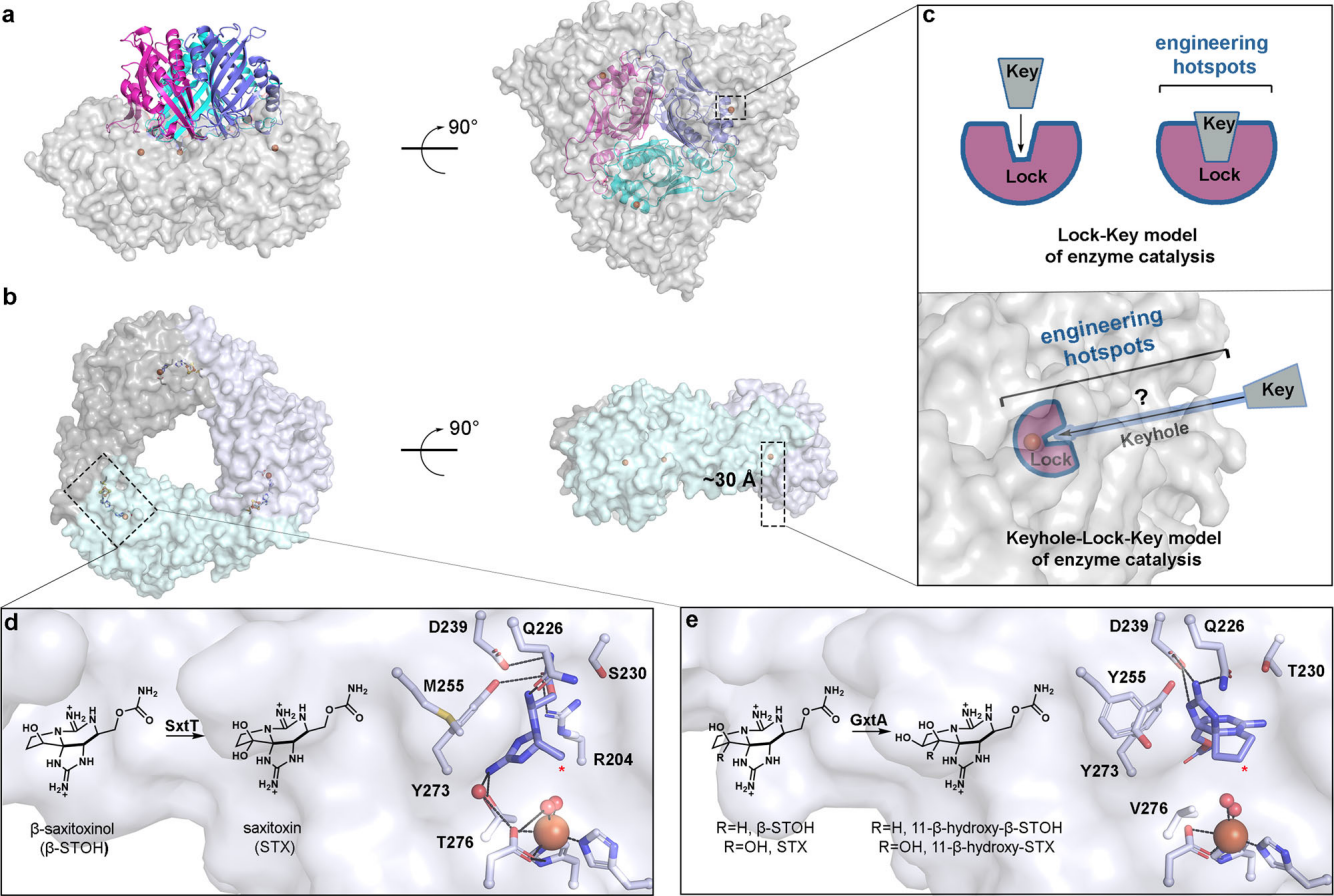
Rieske oxygenases adopt a trimeric architecture that sequesters the metal-based active site and reactive pathway intermediates in the interior of the protein. **a** The quaternary architecture of an α_3_β_3_ Rieske oxygenase that is observed primarily in Rieske oxygenases that catalyze *cis*-dihydroxylation reactions^15–23^. This panel is shown in two orientations that differ by a 90° rotation (PDB: 1NDO^22^). Here, the three catalytic α-subunits are shown in gray and the β-subunits are shown in pink, cyan, and purple. **b** The quaternary architecture of a homotrimeric α_3_ Rieske oxygenase is observed in 10 of the 18 available non-redundant Rieske oxygenase crystal structures^7–14^ (PDB: 6WN3^7^). The active site of the α_3_ Rieske oxygenases is also buried deep within the protein core. **c** The architecture of α_3_β_3_ and α_3_ Rieske oxygenases require the substrate to travel to reach the active site before catalysis can occur. The extra architectural region that the substrate must traverse is known as the “keyhole” and unlike the traditional Lock-Key model of enzyme catalysis, requires the substrate to interact with protein regions outside of the active site^36^. The identity and importance of these auxiliary “keyhole” regions and their relationship to the selectivity of a Rieske oxygenase catalyzed reaction is yet to be determined. **d** SxtT catalyzes the conversion β-saxitoxinol (β-STOH) to saxitoxin (STX) and **e** GxtA catalyzes the conversion of β-STOH and STX to 11-β-hydroxy-β-saxitoxinol and 11-β-hydroxysaxitoxin, respectively^42^. The selectivity exhibited by SxtT and GxtA can be partially attributed to two active site residues that position the substrate correctly for hydroxylation at the respective positions (marked with a red asterisk)^7^.

Rieske non-heme iron oxygenases, or Rieske oxygenases, represent one of Nature’s solutions for performing precise site-selective C–H bond functionalization reactions. This class of enzymes, which consists of more than 80,000 annotated sequences, perform powerful oxidative chemistry with catalyst-controlled selectivity using a combination of a Rieske-type [2Fe–2S] cluster and a mononuclear non-heme iron site^1–5^. The latter metallocenter binds and activates molecular oxygen (O_2_) to facilitate a myriad of reactions, including the monooxygenation, dioxygenation, and oxidative dealkylation reactions for which Rieske oxygenases are recognized^1–6^. Currently, two main architectures have been identified within this enzyme class, a hetero-hexamer that contains three β-subunits of unknown function (α_3_β_3_) and a trimer of the catalytic metallocenter containing α-subunits (α_3_, Fig. 1a, b)^7–23^. In both cases, the active sites are buried deep within the protein interior, a feature shared by many other types of metalloenzymes and more than 60% of proteins^24–27^. These deeply buried active sites serve to protect the reactive metal- and substrate-based intermediates and mitigate the risk of propagating radical chemistry outside of the active site^28,29^. A buried active site in a protein also necessitates a substrate entrance tunnel^25,26,30,31^. Such tunnels have been suggested to facilitate the correct order of multi-substrate reactions^24^. In Rieske oxygenases, this feature may also help ensure that O_2_ binding is coupled to substrate binding and oxidation, which is mainly governed by the oxidation states of the metallocenters and changes to the mononuclear iron site upon substrate binding^3,4,13,24,32–35^.

In line with the keyhole–lock–key model of enzyme catalysis, the substrate entrance tunnel provides extra protein structure between bulk solvent and the active site and has been proposed to play a key role in determining the substrate scope of the Rieske oxygenase naphthalene dioxygenase (Fig. 1a, c)^24,36^. The site-selectivity of a few Rieske oxygenase catalyzed reactions has also been shown to rely on a flexible loop region located in the C-terminal non-heme iron-binding domain of the catalytic Rieske oxygenase α-subunit^8,37–39^. Notably, unlike what is suggested by the traditional lock–key model of enzyme catalysis, which emphasizes only the interactions formed between the substrate and the active site, the so-called “keyhole”, or auxiliary tunnel and loop regions that interact with a substrate, are emerging as enzyme engineering hotspots, mainly recognized for their potential influence on protein stability, substrate scope, and reaction selectivity (Fig. 1c)^30,31,36,40,41^. Unfortunately, an overall lack of structure–function information coupled with low sequence identity between annotated Rieske oxygenases has left many open questions regarding how such auxiliary regions contribute to catalysis.

To investigate the architectural parameters that dictate the substrate specificity and site-selectivity of a Rieske oxygenase catalyzed reaction, we focused on two Rieske oxygenases, SxtT and GxtA, which are involved in the biosynthesis of paralytic shellfish toxins. These enzymes share 88% sequence identity with one another and selectively install a hydroxyl group on adjacent C12 and C11 positions of a tricyclic saxitoxin scaffold, respectively (Fig. 1d, e and Supplementary Fig. 1)^42^. We previously found that the differences between the SxtT and GxtA mononuclear iron-containing active sites are limited to just two residues that play a minor role in controlling C11 versus C12 hydroxylation^7^. In particular, in SxtT, these residues correspond to Met255 and Thr276, which sterically orient and hydrogen bond with the substrate analog dideoxysaxitoxin (ddSTX, Fig. 1d)^7^. In GxtA, these positions instead correspond to Tyr255 and Val276, neither of which interact with ddSTX (Fig. 1e)^7^. Nevertheless, in GxtA, ddSTX undergoes a dramatic rotation in the active site that places C11 as the closest atom to the non-heme iron center^7^ (Fig. 1e). Despite the noted importance of these residues for orienting ddSTX in the active sites of SxtT and GxtA, the activity of these enzymes is not inverted by creating double active site variants. Instead, each double variant remains biased towards its native site-selectivity and substrate^7^. Thus, in this work, we undertook studies to determine the design principles outside of the active site that control the site-selectivity of the SxtT and GxtA catalyzed reactions.

Towards this end, we used X-ray crystallography and Xenon (Xe)-pressurization experiments to identify global differences in the SxtT and GxtA architectures and establish the route of substrate access to the active site. Coupled with site-directed mutagenesis and activity assays, these experiments demonstrated that SxtT and GxtA dictate the site-selectivity of their reactions through a coordinated interplay of three protein regions located inside and outside of the active site. Through the change of only six residues, SxtT, like GxtA, can be made to primarily catalyze hydroxylation at the C11, rather than the C12 position on the tricyclic scaffold and demonstrate a preference for a saxitoxin (STX) substrate rather than a native β-saxitoxinol (β-STOH) substrate. Importantly, we also determined that the implicated protein regions involved in dictating selectivity are conserved in other structurally characterized Rieske oxygenases, suggesting their control over selectivity may be widespread in this enzyme class. Thus, this work outlines the design principles for engineering Rieske oxygenases to have improved activity, broader substrate scope, or altered reaction specificity.

## Results

### Native substrate-bound structures reveal the role of active site residue Tyr255 in selectivity

**Fig. 2:**
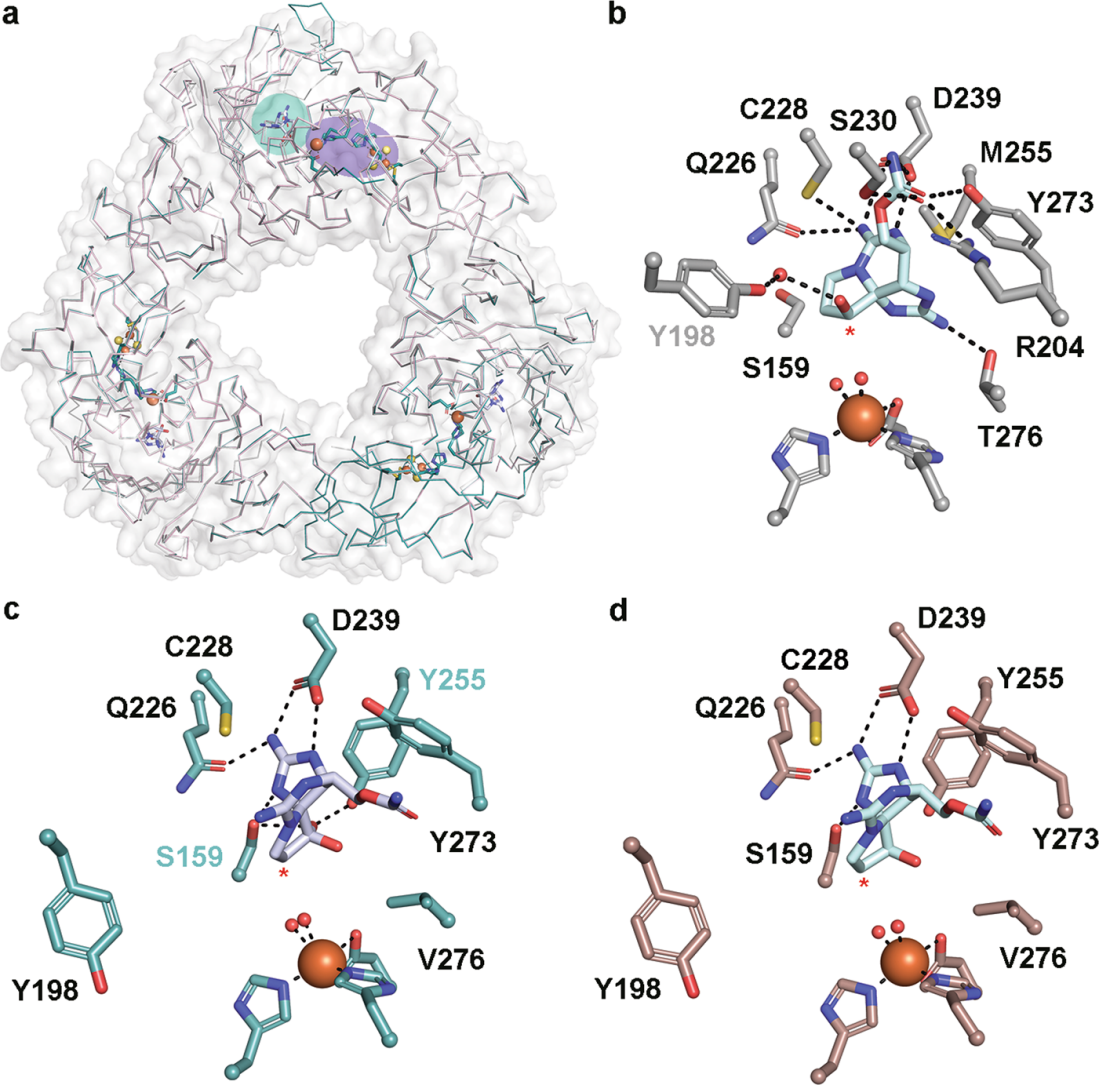
The structures of SxtT and GxtA with their native hydroxylated substrates bound reveal the essential active site interactions. **a** The quaternary trimeric architectures of each determined structure are shown as ribbon diagrams and overlaid with each other (SxtT with β-STOH bound is shown in gray, GxtA with β-STOH bound is shown in pink, and GxtA with STX bound is shown in teal). The metallocenters that bridge two subunits are highlighted in purple and the substrate-binding site of one subunit is highlighted in teal. **b** The structure of SxtT with β-STOH bound reveals the myriad of interactions that this molecule forms in the active site. Tyr198 (gray font) interacts with the β-hydroxyl group of β-STOH via a water molecule. **c** STX binds in the active site of GxtA. The α-hydroxyl group interacts with active site residues Ser159 and Tyr255 (cyan font). **d** Despite the similar makeup of the GxtA active site relative to SxtT, β-STOH binds in a remarkably different orientation and interacts with conserved residues Gln226 and Asp239. In all panels, the position of hydroxylation is marked with a red asterisk.

To investigate how the native hydroxylated substrates bind to SxtT and GxtA and to identify global architectural differences between these proteins, we solved the structures of SxtT and GxtA with β-STOH bound, as well as that of GxtA with STX, bound to 1.79-, 1.79-, and 1.74-Å resolution, respectively (Fig. 2, Supplementary Figs. 2 and 3 and Supplementary Tables 1 and 2). Each structure showcases an α_3_ trimeric architecture, and each protomer of the trimer contains a Rieske cluster in the N-terminal domain and a non-heme iron center in the C-terminal domain (Fig. 2a, Supplementary Fig. 4). The trimeric quaternary architecture places these metallocenters within electron transfer distance of one another across the subunit-subunit interfaces (Fig. 2a, Supplementary Fig. 4). In SxtT, the presumed native substrate β-STOH shows slight deviations in its position in the active site relative to that observed with ddSTX, but it maintains interactions with Arg204, Cys228, Gln226, Ser230, Asp239, Tyr273, and Thr276 (Fig. 1d and Fig. 2b). The differentiating feature between β-STOH and ddSTX, the C12 β-hydroxyl group, forms only one additional interaction in the SxtT active site with Tyr198 (Fig. 2b). This interaction is mediated by a water molecule and Tyr198, like Cys228, Asp239, and Tyr273 is conserved in GxtA. In GxtA, STX also forms extra active-site interactions (Fig. 2c). Here, the addition of a hydroxyl group at the C12 α-position allows for the formation of hydrogen bonds with Ser159 and Tyr255, explaining the previously noted structural importance of Tyr255 for selectivity^7^. The remaining GxtA interactions are formed between β-STOH and STX with Asp239 and Gln226 (Fig. 2c, d).

**Fig. 3:**
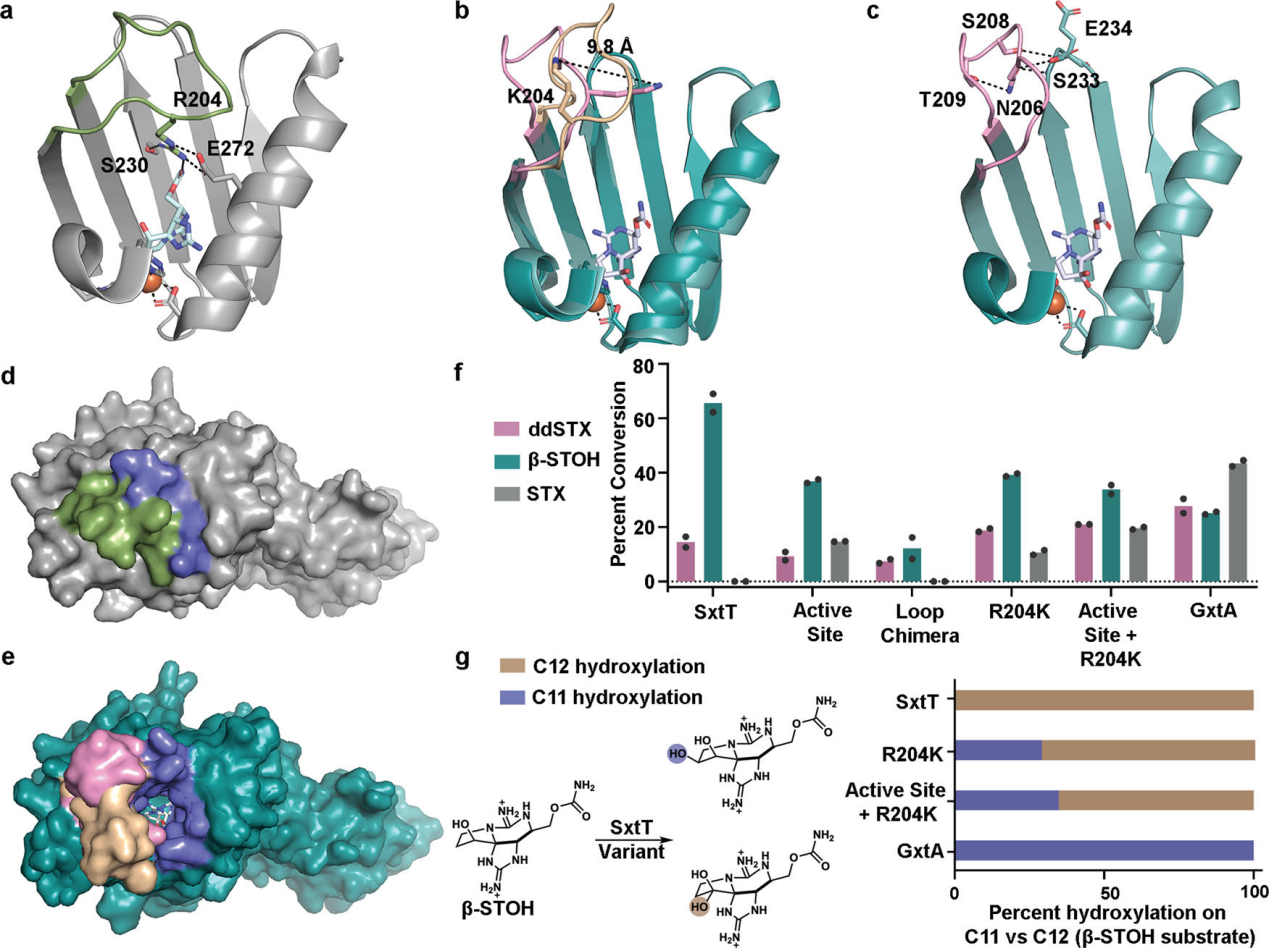
Identification of a second structural region in SxtT and GxtA that is involved in selectivity. Different conformations of a flexible loop that connects the β13 and β14 strands of **a** SxtT and **b** GxtA are observed in each substrate-bound structure. In SxtT this loop (green) interacts with the substrate as well as Ser230 and Glu272. In GxtA, this flexible loop is found in two different orientations (pink and wheat). The difference between the position of Lys204 on these loops is 9.8 Å. **c** The pink loop orientation shown in panel **b** interacts with residues Ser233 and Glu234 on the surface of the protein. **d** A surface rendering of SxtT with β-STOH bound shows that the loop (green) closes the entry (blue) into the active site. **e** A similar surface rendering of GxtA reveals that the different observed loop orientations (pink and wheat) extend away from the active site and leave the entrance (blue) wide open. **f** A chimeric variant in the loop region of SxtT (F200V, Q201H, R204K, I205F, V206N, S207N, H208S, I209T, E210K, and W214M) shows low levels of activity on ddSTX and β-STOH, whereas the single R204K loop variant is able to hydroxylate ddSTX, β-STOH, and STX. Similarly, the creation of a triple variant that combines residue changes at the R204 position with changes in the active site region (M255Y and T276V) amplifies the ability to hydroxylate STX. **g** As evidenced by incorporation of ethanol into the reaction product, a mixture of hydroxylation at the C11 (purple) and C12 (brown) positions is observed with the R204K and M255Y/T276V/R204K variants when β-STOH is used as a substrate. In panels **f** and **g**, data were measured using *n* = 2 independent experiments. In panel **g**, data are presented as the mean value of the measurements. In all panels, ddSTX corresponds to dideoxysaxitoxin, β-STOH corresponds to β-saxitoxinol, and STX corresponds to saxitoxin. Source data are provided as a Source Data file.

The most significant structural difference between these substrate-bound structures of SxtT and GxtA is the orientation of a loop that connects the β13 and β14 strands of the C-terminal iron-binding domain. This loop, which is composed of residues 200–214 shows low sequence identity between the proteins (4 out of 15 residues are conserved) and is flexible, as evidenced by the higher-than-average B-factors in this region of the protein (Supplementary Figs. 1 and 5). The flexibility of this loop region means that for several subunits of both SxtT and GxtA, this loop region was not able to be built. However, in one subunit of SxtT and in two subunits of GxtA, this loop is ordered. In SxtT, this loop extends toward the active site and interacts with β-STOH as well as with non-conserved residues Ser230 and Glu272 (Fig. 3a). In contrast, the two-loop orientations in GxtA extend away from the active site. In one of these orientations, the loop is stabilized by crystal packing (Fig. 3b, Supplementary Fig. 6), whereas in the second orientation, loop residues Asn206, Ser208, and Thr209 interact with the backbone and sidechain of Ser233 and the backbone of Glu234 on the surface of the protein (Fig. 3c). Two of the corresponding loop residues in SxtT (Val206 and Ile209) are hydrophobic and unlikely to reside on the protein surface or form similar interactions with the corresponding surface residues Pro233 and Glu234. Accordingly, whereas both orientations of the GxtA loops reveal an open path from the surface of the protein to the substrate-binding site, the SxtT loop, which is tucked into the protein interior, blocks the analogous path in the crystal structure (Fig. 3d, e).

### Identification of a flexible loop residue that is involved in selectivity

To determine whether the noted differences in sequence and orientation of the flexible loop contribute to the selectivity of the catalyzed reactions, site-directed mutagenesis was used to create a full SxtT chimeric loop variant (F200V/Q201H/R204K/I205F/V206N/S207N/H208S/ I209T/E210K/W214M), a series of shorter chimeric loop variants (F200V/Q201H/R204K, F200V/Q201H/R204K/I205F/V206N/S207N, and F200V/Q201H/R204K/I205F/V206N/S207N/H208S/I209T/E210K), and a single SxtT loop variant at the 204 position (R204K) (Supplementary Fig. 7, Supplementary Table 3). This single variant was chosen based on the observed interaction between this residue and the carbamate sidechain in the structures of ddSTX and β-STOH bound-SxtT (Figs. 1d and 2b). The activity of these variants was tested using VanB as the reductase and ddSTX, β-STOH, and STX as substrates. To our surprise, liquid chromatography–mass spectrometry (LC–MS) experiments showed that the full chimeric loop variant of SxtT has only a low level of activity on ddSTX and β-STOH and no activity on STX (Fig. 3f and Supplementary Fig. 8). The observed lack of hydroxylation on STX suggests the selectivity is unchanged in the chimeric loop SxtT variant. However, to ensure that this lack of activity is due to changes in the primary structure of the loop region, rather than changes to the folding of the variant relative to wild-type SxtT, a circular dichroism (CD) experiment was used to demonstrate that the full chimeric loop variant is similar in secondary structure to wild-type SxtT (Supplementary Fig. 9a).

In contrast to the chimeric loop variant, the R204K-SxtT variant shows activity on each of the three provided substrates (Fig. 3f and Supplementary Fig. 8). To identify the specific residue that leads to the drastic difference in activity between the single and full loop variants, the ability of the shorter chimeric loop variants to hydroxylate ddSTX, β-STOH, and STX, was probed. Here, it was determined that each tested variant showed decreased activity on ddSTX and β-STOH relative to wild-type SxtT (Supplementary Fig. 10). Only the R204K- and F200V/Q201H/R204K-SxtT loop variants show any activity on STX. As the R204K-SxtT variants show the highest level of activity on STX, a study to identify the position of hydroxylation when provided a β-STOH substrate was undertaken. Here, we capitalized on a previously established method to distinguish between hydroxylation at the C11 and C12 positions^7^. This method, which relies on the reaction of one molecule of ethanol with STX to form a hemiketal, reveals when hydroxylation has occurred at the C12 position (Supplementary Fig. 11a). This experiment demonstrated that the R204K-SxtT variant, like the M255Y/T276V double active site variant of SxtT, produces both an ethanol-incorporated product and a non-ethanol incorporated product, suggesting that this variant displays some degree of altered site-selectivity relative to the wild-type enzyme (Fig. 3g and Supplementary Fig. 11). This R204K SxtT variant shows less C12 hydroxylation than the double active site variant, but the selectivity remains biased toward the native site-selectivity (Fig. 3g and Supplementary Fig. 11). This finding prompted an investigation into whether a triple variant (M255Y/T276V/R204K) that combines changes in the active site and the loop would show an increased affinity for hydroxylating at C11. Indeed, the combination of the active site substitutions with the R204K loop substitution shows an increase in the ability to hydroxylate STX and an approximate nine-percent increase in the amount of hydroxylation at C11 relative to the R204K variant, suggesting that the flexible loop region and active site integrate to confer selectivity in SxtT and GxtA (Fig. 3f, g and Supplementary Figs. 8 and 11). However, these results also show that these two structural regions are not enough to fully account for the selectivity differences between SxtT and GxtA.

### Identification of a substrate entrance tunnel that is important for selectivity

**Fig. 4:**
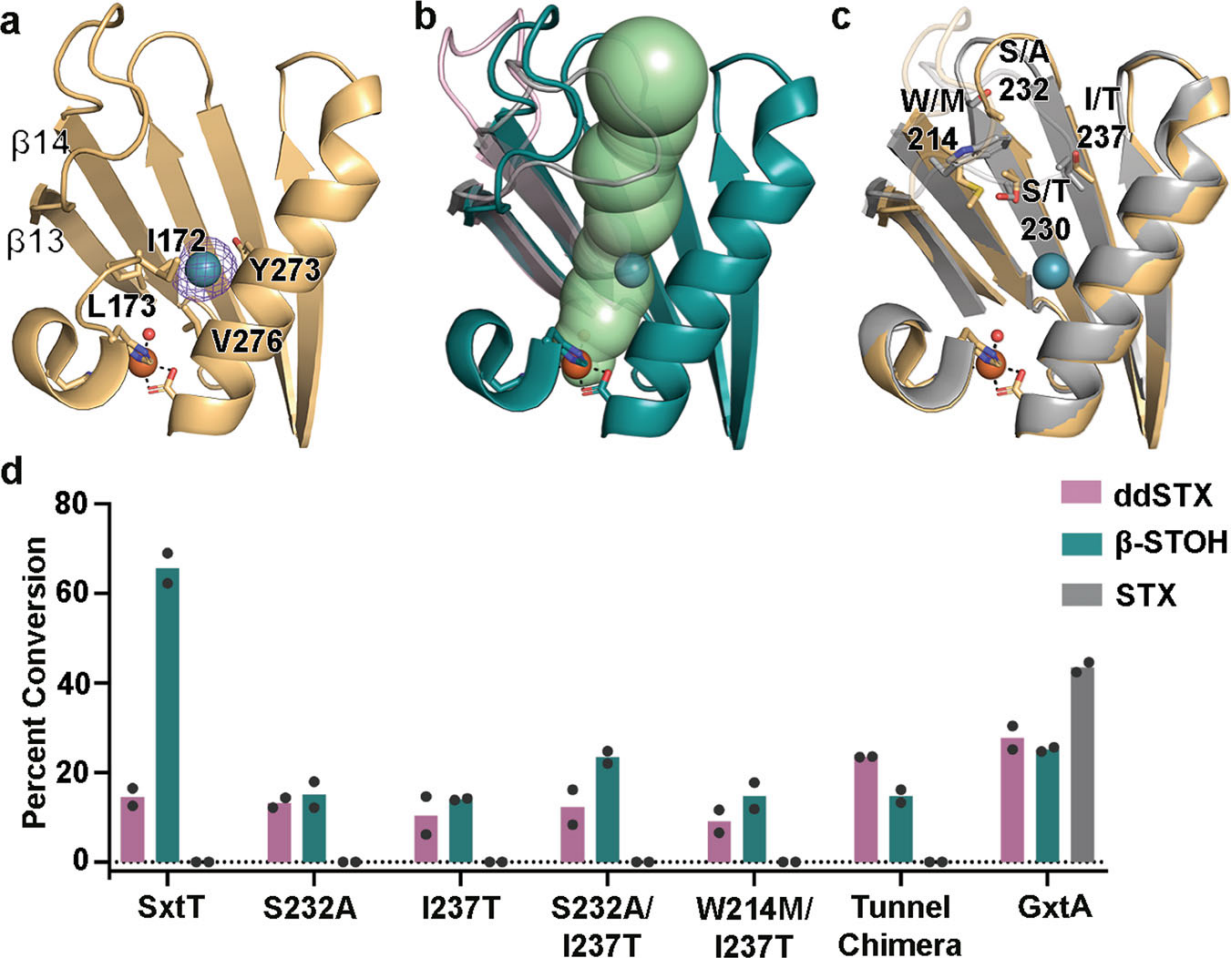
The route of substrate entry into the active site was visualized using crystals of GxtA that were pressurized with Xenon (Xe). **a** A Xe-pressurized structure of GxtA shows the presence of Xe near the mononuclear iron-containing active site. The location of Xe was verified by calculation of anomalous maps, which are shown in purple mesh and contoured around Xe at 3.0*σ*. **b** An approximate 33-Å long tunnel connects the surface of GxtA to the non-heme iron-containing active site. This calculated tunnel overlaps with the position of Xe. The loops of GxtA (pink and teal) leave this tunnel open, whereas the loop of SxtT (gray) closes off this path. **c** The SxtT (gray) and GxtA (golden yellow) regions around the tunnel differ at several positions. The flexible loops are shown at low transparency to show the differences more clearly in tunnel lining residues. **d** Only low levels of activity are observed when ddSTX and β-STOH are combined with single (S232A, I237T), double (S232A/I237T, W214M/I237T), and chimeric (W214M/S232A/I237T) SxtT tunnel variants. Of note, there is no activity on STX using SxtT or any tunnel variant tested. In panel d, data was measured using *n* = 2 independent experiments. In all panels, ddSTX corresponds to dideoxysaxitoxin, β-STOH corresponds to β-saxitoxinol, and STX corresponds to saxitoxin. Source data are provided as a Source Data file.

Based upon the success of identifying a residue in the flexible loop that is involved in site selectivity, we experimentally probed the location of a substrate entrance tunnel to the active site. Crystals of SxtT and GxtA were pressurized with Xe, cryo-cooled, and placed in the X-ray beam. A resultant 2.69-Å resolution Xe-pressurized crystal structure of GxtA revealed the presence of Xe in each protomer of the trimeric architecture (Fig. 4a). The Xe binding sites were confirmed by calculation of anomalous maps (Fig. 4a). Each Xe is located at the base of a computationally predicted tunnel for transporting substrate into the active site of GxtA^7^. This tunnel is impassable by the loop orientation in SxtT but is wide-open in GxtA, as reported previously^7^ (Fig. 4b). The Xe binding site is formed mainly by hydrophobic residues Val276, Leu173, Ile172, as well as Tyr273 (Fig. 4a). Confirmation of the substrate entrance tunnel prompted calculation of an analogous tunnel in SxtT using the MOLEonline server^43,44^. This task required the removal of Arg204 from the structure prior to calculation. In both proteins, the tunnels span a similar distance of approximately 33 Å. The major differences between the corresponding tunnels are the residues located at the 214 (bridges the loop and tunnel), 230, 232, and 237 positions, the overall tunnel hydrophobicity, and the width of the tunnels (Fig. 4c and Supplementary Fig. 12). In particular, the GxtA tunnel showcases a higher hydropathy index than that of the tunnel found in SxtT and a larger bottleneck radius of 2.7 Å (Supplementary Fig. 12). This tunnel width is mainly limited by residues in the center of the tunnel (Thr230, Thr237, and Tyr273), whereas the smaller 1.7 Å tunnel radius observed in SxtT is limited by residues located near the entrance (Ser230, Ile237, and Glu272, Supplementary Fig. 12).

Using the experimentally confirmed substrate access tunnel as a guide, we tested the importance of tunnel lining and bottleneck residues in dictating the site selectivity of the SxtT catalyzed reaction using site-directed mutagenesis experiments (Supplementary Fig. 7, Supplementary Table 3, and Fig. 4d). Targeted residues in SxtT (Trp214, Ser232, and Ile237) included those that are larger or differ in polarity from the corresponding residues in GxtA (Met214, Ala232, and Thr237). Again, as described for the SxtT loop variants, to determine whether these tunnel variants showed changes in site-selectivity, we tested their activity using VanB as an electron donor and ddSTX, β-STOH, and STX as substrates. Here, LC–MS experiments showed that single (I237T, S232A), double (S232A/I237T and W214M/I237T), and chimeric (W214M/S232A/I237T) tunnel variants, despite showing similar CD spectra to wild-type SxtT, show no activity on STX and lower amounts of activity on ddSTX and β-STOH than GxtA (Fig. 4d and Supplementary Figs. 9b and 13).

### Coordinated interplay of protein regions dictates substrate-specificity and site-selectivity

**Fig. 5:**
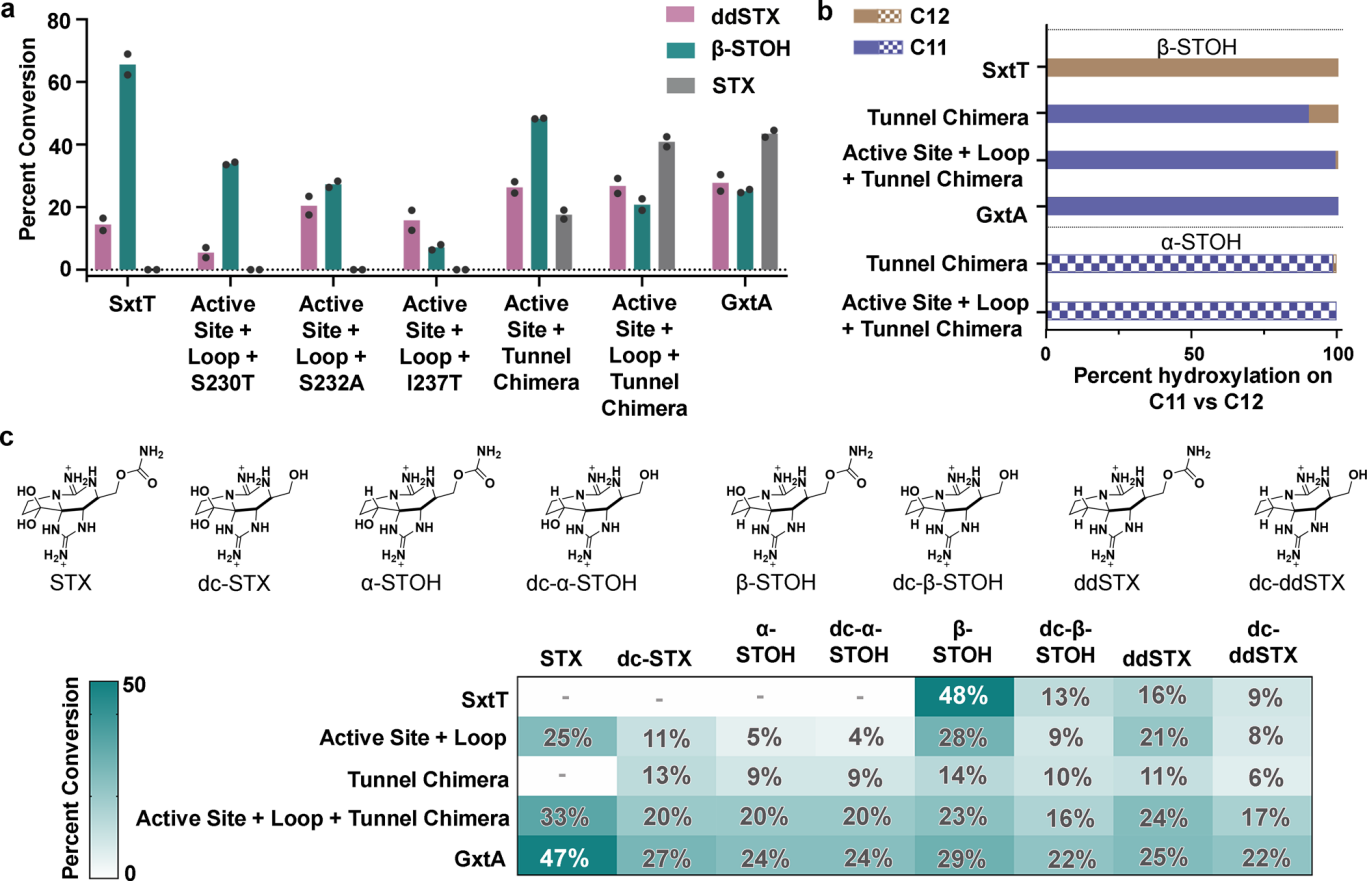
A combination of residues from the active site, loop, and tunnel region are responsible for the selectivity and substrate scope of the SxtT catalyzed reaction. **a** The effect of combining residue variants from the active site (M255Y, T276V), loop (R204K), and tunnel region (W214M/S232A/I237T) was tested using ddSTX, β-STOH, and STX as substrates. **b** The position of hydroxylation on a β-STOH substrate was confirmed using an ethanol-incorporation experiment. The essential lack of an ethanol-derivatized product in the active site + loop + tunnel chimera variant (99% of hydroxylation at C11) suggested that selectivity had been changed to resemble GxtA (solid colors). The position of hydroxylation when α-STOH was used as a substrate was tested in the tunnel chimera and the active site + loop + tunnel chimera variants (patterned colors). Both variants, like GxtA, showed a preference for hydroxylating at the C11 position. In the tunnel chimera, using an α-STOH substrate, 98% of the product is hydroxylated at the C11 position, whereas in the active site + loop + tunnel chimera variant, 100% of the product showcases a hydroxyl group at the C11 position. **c** The ability to hydroxylate naturally occurring saxitoxin analogs (top panel) was evaluated for the active site + loop, tunnel chimera, and the active site + loop + tunnel chimera variants. The activity is shown as a heat map with the lowest activity in white and the highest activity in dark teal. The percent conversion of the native substrates of SxtT and GxtA are shown in white. In panel a, data was measured using *n* = 2 independent experiments. In panels **b** and **c**, the data were also measured using *n* = 2 independent experiments and is presented as the mean value of these measurements. In panels **a** and **c**, ddSTX corresponds to dideoxysaxitoxin, β/α-STOH corresponds to β/α-saxitoxinol, and STX corresponds to saxitoxin. The abbreviation dc-represents decarbamoyl. Source data are provided as a Source Data file.

As additively varying the sequences in the active site and loop regions led to an increased ability to hydroxylate at C11, the effect of combining changes in the tunnel, active site, and loop regions was subsequently tested. First, individual tunnel residue mutations were introduced into the construct that contains two mutations in the active site and one on the loop to produce M255Y/T276V/R204K/S230T, M255Y/T276V/R204K/S232A, and M255Y/T276V/R204K/I237T variants (Supplementary Fig. 7 and Supplementary Table 3). These new variants, which were confirmed to be properly folded by CD, still lacked the propensity to hydroxylate STX (Fig. 5a, Supplementary Fig. 9b, Supplementary Fig. 14). However, when we cumulatively changed three tunnel lining residues and two active site residues (M255Y/T276V/W214M/S232A/I237T), activity was detected on ddSTX, β-STOH, and STX (Fig. 5a, Supplementary Fig. 14). Introduction of the R204K loop mutation into this construct (M255Y/T276V/W214M/S232A/I237T/R204K) showed, for the first time, the highest level of activity with the STX substrate, suggesting that the selectivity of SxtT had been inverted to resemble GxtA (Fig. 5a, Supplementary Fig. 14). The ability to catalyze hydroxylation at C11 in this latter variant was confirmed by looking for ethanol incorporation in the reaction that used β-STOH as a substrate (Fig. 5b and Supplementary Fig. 15). In this case, the essential lack of identification of an ethanol-incorporated product confirmed that 99% of hydroxylation occurred at the C11, rather than the C12 position on the substrate (Fig. 5b). These results suggest that the flexible loop region, the tunnel, and the active site integrate to confer selectivity and specificity in SxtT and GxtA.

Intriguingly, our structures did not reveal any interactions that were formed between tunnel lining residues and substrate in SxtT or GxtA. Thus, we hypothesize that the tunnel is involved in transporting and handing off the substrate to the active site in the correct orientation for hydroxylation. Commensurate with this hypothesis, 90% of the hydroxylated product made by the SxtT tunnel chimera variant (W214M/S232A/I237T), when β-STOH was provided as a substrate, was hydroxylated at the C11 position (Fig. 5b, Supplementary Fig. 15).

### Identification of the architectural parameters that dictate substrate scope

The altered selectivity of the SxtT tunnel chimera variant and the ability of the active site-tunnel-loop variant (M255Y/T276V/W214M/S232A/I237T/R204K) to hydroxylate STX inspired an investigation into whether these variants, like GxtA^42^, would also hydroxylate other STX-derived compounds, including α-STOH, decarbamoyl (dc)-α-STOH, dc-STX, ddSTX, and dc-ddSTX (Fig. 5c). The three α-hydroxyl group-containing STX analogs (α-STOH, dc-α-STOH, dc-STX) are not accepted by wild-type SxtT. However, as described for STX, when VanB was used as a reductase and LC–MS was used to assess hydroxylation, the M255Y/T276V/R204K, W214M/S232A/I237T, and M255Y/T276V/R204K/W214M/S232A/I237T variants show an increasing ability to hydroxylate these compounds (Fig. 5c and Supplementary Fig. 16). In particular, as previously described for wild-type GxtA, the tunnel chimera variant (W214M/S232A/I237T) catalyzes hydroxylation on α-STOH at the C11 position (Fig. 5b, Supplementary Fig. 17). Again, this data suggests that the tunnel is important for transporting and placing the substrate in the correct orientation for hydroxylation in the active site. Intriguingly, despite the noted ability of the tunnel chimera variant to accept substrates with an α-hydroxyl group, it does not hydroxylate STX. To accomplish this feat, the variant that also showcases changes in the active site and loop region (M255Y/T276V/R204K/W214M/S232A/I237T) is required (Fig. 5b, Supplementary Fig. 15). Like wild-type GxtA, this variant also hydroxylates α-STOH at the C11 position (Fig. 5b, Supplementary Fig. 17).

As expected for wild-type SxtT, the highest amount of activity is observed on a β-STOH substrate (Fig. 5c). Correspondingly, activity on this native substrate decreases as SxtT is mutated to resemble GxtA. For ddSTX, dc-ddSTX, and dc-β-STOH, each of which has been previously shown to be hydroxylated by both wild-type enzymes^42^, the highest level of activity is observed with wild-type GxtA (Fig. 5c). For ddSTX, dc-ddSTX, and dc-β-STOH, consistently low levels of activity are encountered in the W214M/S232A/I237T variant, whereas activity is amplified to resemble wild-type GxtA by also changing the active site and loop regions (Fig. 5c, Supplementary Figs. 13, 14, and 16). Collectively, these results confirm that the substrate specificity and selectivity of SxtT can be changed to mirror that of GxtA by changing just six residues that span the active site, tunnel, and loop regions of the protein.

### Identification of conserved loop and tunnel regions in additional Rieske oxygenases

**Fig. 6:**
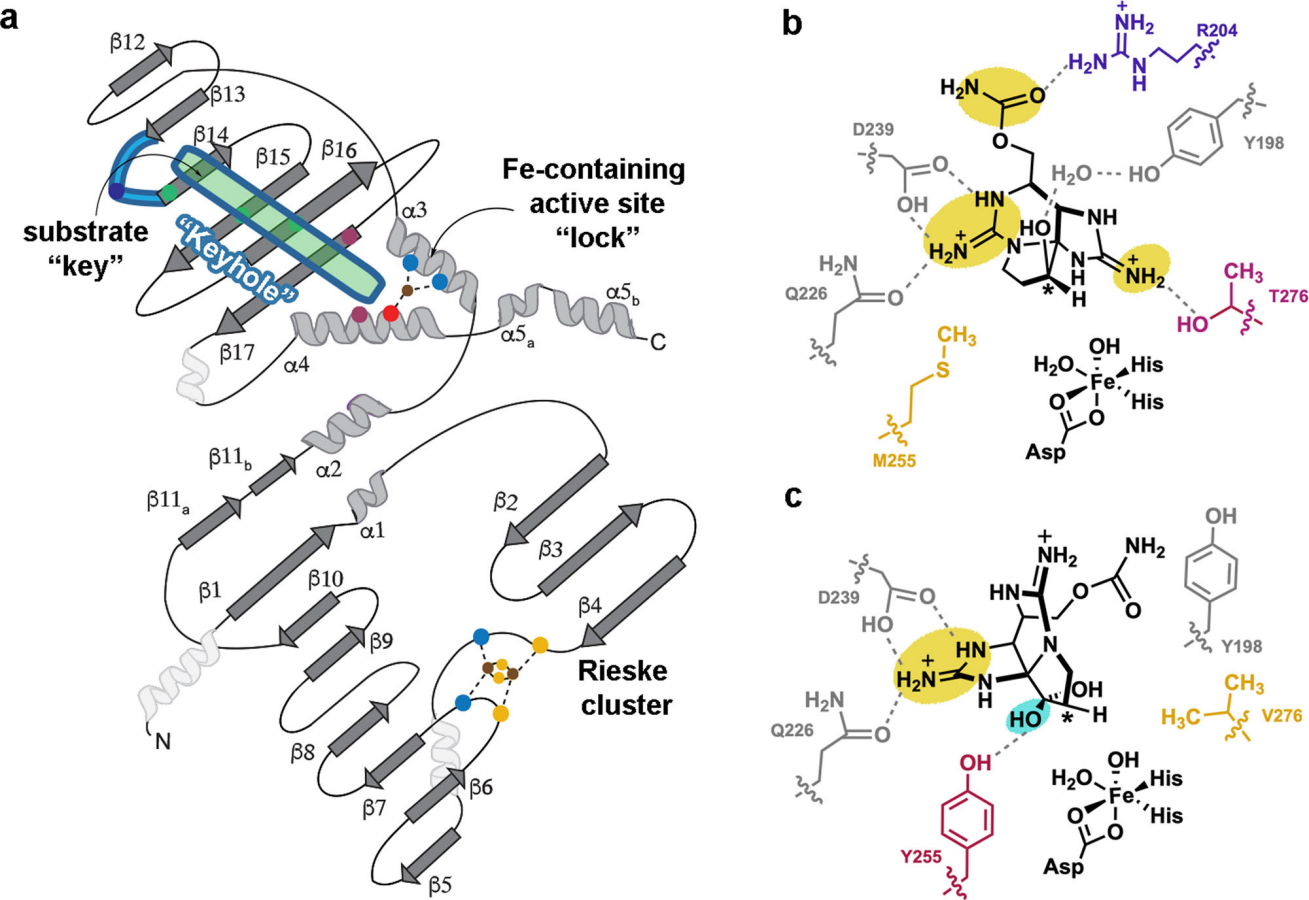
A coordinated interplay of three protein regions in SxtT is important for dictating selectivity. **a** A topology diagram of SxtT/GxtA. The active site (dark pink circles represent residues 255 and 276), loop region (light blue, the dark blue circle represents residue 204) and tunnel (green, green circles represent residues at the 214, 232, and 237 positions) each have residues that are involved in dictating selectivity. The so-called “keyhole” (loop and tunnel region) of these proteins is outlined in blue. The secondary structure that is conserved among the structurally characterized α_3_ Rieske oxygenases is shown in dark gray and light gray^5^. The three helices in white are not conserved. **b** Three regions of β-STOH that are also found in STX are recognized by SxtT (yellow): the carbamate is recognized by Arg204 from the flexible loop and the guanidium ions are recognized by active site residues Thr276, Gln226, and Asp239. Of these residues, only Thr276 and Arg204 are unique to SxtT. **c** Two regions of STX are recognized by active site residues in GxtA: the guanidinium moiety of the five-membered ring interacts with Gln226 and Asp239 and the α-hydroxyl interacts with Tyr255. Of these recognition points, only the α-hydroxyl (teal) is unique to STX and only Tyr255 is unique to GxtA.

The parallels that can be drawn between SxtT, GxtA, and other Rieske oxygenases make it tempting to consider whether these design principles are conserved in other enzymes of this class. Indeed, our results on the M255Y/T276V/R204K variant, which shows a pronounced ability to hydroxylate STX, are reminiscent of what is observed in the caffeine demethylating Rieske oxygenases NdmA and NdmB^8^. In this system, replacement of NdmA loop with the corresponding loop residues from NdmB, coupled with changes in the active site, results in altered *N*-demethylase activity^8^. Structural alignment of these enzymes with SxtT and GxtA reveals that the implicated selectivity loop in NdmA, NdmB, SxtT, and GxtA, similarly occurs between the β13 and β14 strands (Supplementary Fig. 18a, b). To determine whether the flexible loop and tunnel are more common among Rieske oxygenases, we analyzed the B-factors of the additional six other structurally characterized α_3_-oxygenases (Supplementary Fig. 18). These structurally characterized enzymes, include those that are involved in *N*-demethylation of *N,N-*dimethylproline (Stc2)^12^, as well as those that are responsible for the degradation of cholesterol (KshA)^10^, carnitine (CntA)^11^, 2-oxoquinoline (OMO)^13^, dicamba (DdmC)^9^, and carbazole (CARDO)^14,45^. Through analysis of the structures’ B-factors, a loop of high flexibility that exists between the β13 and β14 strands of the C-terminal domain, analogous to that observed in SxtT and GxtA, can be identified in the structures of NdmA, NdmB, KshA, CntA, and DdmC (Supplementary Fig. 18 and Fig. 6a). Consistently, the loop implicated in NdmA and NdmB selectivity is so flexible that it was not modeled into the crystal structures^8^ (Supplementary Fig. 18b). Similarly, the flexible loop region in KshA is partially missing and has been shown to play a role in dictating the substrate preference of KshA homologs^10,38^ (Supplementary Fig. 18c). A disordered and partially missing loop is also observed in the apo- and substrate-bound structures of CntA^11^ and in DdmC^9^ (Supplementary Fig. 18d, e). Importantly, in these five Rieske oxygenases a tunnel that leads from this loop region to the mononuclear iron site could be calculated using the MOLEonline server^43,44^ (Supplementary Fig. 19). In all cases, this tunnel traverses across the same secondary structure seen in SxtT and GxtA and is oriented similarly with respect to the flexible loop (Supplementary Fig. 19 and Fig. 6a). In Stc2, there is flexibility in the analogous loop, but based on its secondary structure which folds in towards the substrate-bound in the active site, an active site tunnel is not obvious, nor can it be calculated (Supplementary Fig. 18f). In CARDO, a tunnel was also not observed, presumably due to the region that connects β13 and β14 being much longer and containing an extra β-strand and β-hairpin relative to its counterparts in SxtT and GxtA. Nevertheless, residues in this region have been suggested to be dynamic and to gate the active site upon substrate binding^45–48^ (Supplementary Fig. 18g). Finally, in OMO, an ordered, mainly hydrophobic loop that resembles the long loop in CARDO similarly precludes identification of an active site tunnel (Supplementary Fig. 18h). Importantly, however, an adjacent region in the tertiary architecture of OMO has been implicated in substrate channeling to the active site^13^. Thus, these results suggest the possibility that like SxtT and GxtA, these other structurally characterized Rieske oxygenases will employ similar architectural features, including a loop, tunnel, and active site to recognize and orient a substrate for site-selective chemistry.

## Discussion

Here, we elaborated on the design principles that dictate how the Rieske oxygenases SxtT and GxtA catalyze site-selective monohydroxylation reactions. In particular, the specificity and selectivity of these enzymes require a coordinated interplay of three protein regions: the active site, a flexible loop, and the substrate entrance channel (Fig. 5 and Fig. 6a). Structurally it was shown that the native hydroxylated substrates of SxtT and GxtA bind in the active site similarly to ddSTX, albeit with some important key differences. Namely, β-STOH in SxtT makes a through-water interaction with active site residue Tyr198 and STX in GxtA interacts with Tyr155 (Fig. 2).

A comparison of these structures and those previously determined^7^ also revealed important orientations of a flexible loop that can serve as a gate to the active site by assuming open (GxtA) or closed states (SxtT, Fig. 3a–e). The importance of this loop is demonstrated by the chimeric loop variants described in this work, which show that changing the SxtT loop to resemble the GxtA counterpart results in lower levels of activity ddSTX and β-STOH (Fig. 3f and Supplementary Fig. 10). Outside of considering the overall loop architecture, in SxtT, the loop is important as it interacts with a bound substrate using residue Arg204 (Figs. 1d and 2b). This Arg204 interaction, coupled with additional interactions that are formed between active site residues (Thr276, Gln226, Ser230, Asp239, and Tyr273) and the guanidinium groups of the five- and six-membered rings of β-STOH, ensures that three regions of ddSTX and β-STOH are recognized by SxtT (Fig. 6b). The lower activity of SxtT on ddSTX shown here, and previously noted^42^, is likely correlated to the ability of β-STOH to interact with Tyr198 via a water molecule (Fig. 2b). In GxtA, there are only two recognition points for the native STX substrate. Gln226 and Asp239 interact with the guanidinium of the five-membered ring of ddSTX, β-STOH, and STX, and Tyr255 interacts with the α-hydroxyl group of STX, which is installed by SxtT (Figs. 1e, 2c, d, and Fig. 6c). The conservation of Gln226 and Asp239 between SxtT and GxtA suggests that any conformational freedom afforded in the SxtT active site could allow rotation of the substrate and formation of the interactions that permit hydroxylation at C11 (Fig. 6b, c).

Indeed, in the R204K variant, the SxtT substrate loses one of its two unique interacting residues, and a low level of non-native hydroxylation is observed (Fig. 3f, g). A similar phenomenon is observed in the single and double active site variants of SxtT; loss of Thr276, which interacts with the guanidinium of the five-membered ring in ddSTX and β-STOH, allows SxtT to catalyze non-native hydroxylation at C11^7^. Likewise, loss of interactions with the SxtT active site by removal of the carbamate sidechain from the native substrates would account for the lower levels of activity observed when SxtT is given dc-ddSTX or dc-β-STOH as a substrate (Fig. 5c). Tyr255 in GxtA interacts with the α-hydroxyl group of STX (Fig. 2c). Introduction of this residue in place of Met255 in SxtT in isolation or combined with a T276V and R204K mutation can account for the ability of previously investigated SxtT M255Y and M255Y/T276V variants^7^ as well as the M255Y/T276V/R204K variant to catalyze low levels of hydroxylation on STX and at the C11 position of β-STOH (Fig. 3f, g). Similarly, the presence of Tyr255 is likely the reason that the M255Y/T276V/R204K SxtT variant can accommodate additional substrates that like STX, contain a C12 α-hydroxyl group (α-STOH, dc-α-STOH, and dcSTX) (Fig. 5c and Fig. 6b, c).

As described for the double active site SxtT variant^7^, changing just the active site and loop residue Arg204 to resemble GxtA adds functionality to SxtT as it can now hydroxylate a substrate at either the C11 or C12 position and accommodate a broader range of substrates (Fig. 3f). However, the M255Y/T276V/R204K variant still shows a preference for a β-STOH substrate and hydroxylation at C12 (Fig. 3g). To bestow SxtT both the substrate-specificity and site-selectivity of GxtA require consideration of a third architectural parameter, a tunnel that leads from the surface of the protein into the active site. Indeed, in this work, we demonstrated that changing just three residues in the tunnel of SxtT to mirror GxtA (W214M/S232A/I237T) allows SxtT to preferentially hydroxylate β-STOH at the C11 position (Fig. 5a, b). The W214M/S232A/I237T variant, like the M255Y/T276V/R204K variant, can also hydroxylate α-STOH, dc-α-STOH, and dc-STX substrates, albeit to a greater extent (Fig. 5c). The latter α-STOH substrate, like β-STOH, is preferentially hydroxylated at the C11 position (Fig. 5b). Yet, despite the observed ability of this tunnel variant to catalyze hydroxylation at C11, like wild-type SxtT, it does not accept STX as a substrate. However, when these tunnel residues changes are accompanied by the addition of GxtA active site residues (M255Y/T276V/W214M/S232A/I237T), STX is hydroxylated. Finally, when these tunnel residue changes are accompanied by both changes to the active site and to the loop (M255Y/T276V/R204K/W214M/S232A/I237T), STX conclusively becomes the preferred substrate (Fig. 5a, b). These results indicate that SxtT, can showcase the substrate-specificity and site-selectivity of GxtA by (i) the removal of both unique residues that interact with the native substrate of SxtT (Thr276 and Arg204), (ii) the addition of a residue that interacts with the α-hydroxyl of STX in GxtA (Tyr255), and (iii) the replacement of three SxtT tunnel lining residues with the GxtA counterparts to change the primary site of hydroxylation from C12 to C11. As described in the keyhole-lock-key model of enzyme catalysis^36^, the substrates of these enzymes must be recognized and positioned by the “keyhole”, or the loop and tunnel regions, to be correctly fit into the active site for hydroxylation (Fig. 6a).

To investigate which architectural features of SxtT and GxtA are more prevalent in the Rieske oxygenase family, which contains more than 80,000 annotated Rieske oxygenase sequences and only 18 unique crystal structures^5,7^, we undertook an investigation into whether α_3_ enzymes would showcase similar loop and tunnel regions. We found that in almost all cases, a loop of high flexibility exists in an analogous region to that observed in SxtT and GxtA (Supplementary Fig. 18). Furthermore, for most of the proteins that possess a flexible loop, we can calculate a tunnel to the active site that is comparable to what is seen in SxtT and GxtA (Supplementary Fig. 19). These results suggest the possibility that like SxtT and GxtA, some of these other structurally characterized Rieske oxygenases will employ three architectural regions to orient and position substrate, and to dictate substrate scope. It is also intriguing to consider whether these architectural parameters will be employed in the α_3_β_3_ Rieske oxygenases. Based on recent computational studies performed on Naphthalene 1,2 dioxygenase, that showed the value of using an entrance tunnel to predict substrate scope, we hypothesize the active site tunnels have thus far been underappreciated in studying Rieske oxygenase selectivity^24^. In addition, based on recent work that shows changes to the flexible loop region in cumene dioxygenase gives rise to altered reactivity, substrate scope and reaction selectivity, and may alter the substrate access tunnel^37^, we hypothesize that the cooperativity between the three protein regions in SxtT and GxtA may also be used by the α_3_β_3_ enzymes. Thus, we are excited to explore the possibility that these design principles will be conserved across the Rieske oxygenase class of enzymes and to decipher whether they can be exploited to predictively repurpose and manipulate the diverse catalytic repertoire of Rieske oxygenases.

## Methods

### Site-directed mutagenesis

The Agilent QuickChange Lightning Site-Directed Mutagenesis Kit and Bio-Rad C1000 Thermal Cycler were used to introduce the desired mutations into SxtT. In brief, each 50 µL reaction mixture contained 5 µL of 10× reaction buffer, 1 µL of 2 ng/µL pMCSG-9-SxtT-containing construct, 0.5 µL or 125 ng of each oligonucleotide primer, 1 µL of dNTP mix, 1.5 µL of Agilent QuickSolution reagent, and 1 µL of Agilent QuickChange Lightning Enzyme. Each mutagenesis reaction was subsequently run for 18 cycles (95 °C 20 s, 60 °C 10 s, 68 °C 5 min). Following amplification, the reaction mixtures were digested at 37 °C for 10 min following the addition of 2 µL of DpnI restriction enzyme. Finally, 5 µL of the DpnI digest reaction mixture was transformed into XL10-Gold ultracompetent cells, and mutations were verified by Sanger sequencing (Genewiz). Primer sequences can be found in Supplementary Table 3.

### Protein expression conditions for SxtT, GxtA, and variants

To produce wild-type and variant protein samples for use in X-ray crystallography and activity assays, we capitalized on the expression and purification methods that were used in our previous work^7^. In short, single colonies of C41(DE3) *Escherichia coli* cells containing the *sxtT* or *gxtA* pMCSG9 plasmids were used to inoculate 5 mL of LB containing 100 µg/mL ampicillin. These starter cultures were incubated overnight at 37 °C and subsequently used to inoculate 1 L of LB media containing 100 µg/mL ampicillin. These larger cultures were grown in similar conditions to the starter culture (37 °C and 200 rpm). At the time when the OD_600_ reached 0.6–0.8, the temperature was decreased to 20 °C and 0.2 mM IPTG, 0.2 mg/mL ferric ammonium citrate, and 0.4 mg/mL ferrous sulfate heptahydrate were added. Each of these large-scale cultures was incubated for an additional 18 h prior to harvesting. The average pellet for a 1 L wild-type SxtT culture was 5 g of wet cell mass. For the SxtT tunnel chimera, SxtT loop chimera, and SxtT M255Y/T276V/R204K/S230T, M255Y/T276V/R204K/S232A, and I237T variants, the average pellet mass was 2–2.5 g for 1 L of cell culture. The wet cell mass for all other SxtT variants except for the 5 variants mentioned above was 3.5–4.5 g for 1 L cell culture.

### Purification protocol for SxtT and GxtA for reactions and crystallography

To obtain homogeneous SxtT and GxtA protein for X-ray crystallography and activity assay experiments, a similar protocol to that previously described was followed^7^. Briefly, the cell pellet produced from a 2 L culture that contained the over-expressed MBP-tagged SxtT and GxtA was resuspended in 50 mL of lysis buffer (50 mM Tris-HCl pH 8.0, 1 M NaCl, 10% glycerol). Cells were lysed by sonication using a program that proceeds through repetitive cycles of 4 s sonication and 15 s rest until reaching a total on-time of 5 min. Lysed cells were clarified in an Eppendorf centrifuge 5810R at 12,000 × *g* for 40 min. The supernatant, which contained the MBP-tagged protein SxtT or MBP-tagged GxtA was loaded onto a 5 mL MBP-Trap column (Cytiva). This column was washed with 10 column volumes of Buffer A (50 mM Tris-HCl pH 8.0, 1 M NaCl, 10% glycerol) and eluted with a five-column volume gradient of Buffer B (50 mM Tris-HCl pH 8.0, 1 M NaCl, 10% glycerol, 10 mM maltose). Fractions containing the MBP-SxtT or MBP-GxtA were pooled and dialyzed into Buffer C (20 mM Tris-HCl pH 7.4, 50 mM NaCl, 1 mM DTT, 10% glycerol). Following buffer exchange, the MBP-tagged SxtT and GxtA proteins were cleaved using 5 mg of TEV protease. This tag-free protein was subsequently loaded onto a 5 mL His-Trap (Cytiva) for further purification and removal of both the TEV protease and the cleaved His-MBP tag. The flow-through from the column, which contained the desired tag-free proteins was concentrated and loaded onto a HiPrep 16/60 Sephacryl S200-HR (Cytiva) gel filtration column that was pre-equilibrated in Buffer D (50 mM HEPES, pH 8.0, 200 mM NaCl, 10% glycerol). The trimeric protein fractions were pooled and concentrated to 10 mg/mL. This concentrated protein was aliquoted into 50 µL fractions and flash-frozen using liquid nitrogen for storage in the −80 °C freezer. For crystallography, the trimeric protein fractions were pooled, and buffer exchanged into Buffer E (50 mM HEPES, pH = 8.0, 10% glycerol) using PD-10 desalting columns (BioRad). The cell pellet from a 1 L culture of wild-type SxtT typically results in a yield of ~5 mg of homogenous protein. For the SxtT tunnel chimera, SxtT loop chimera, and SxtT M255Y/T276V/R204K/S230T, M255Y/T276V/R204K/S232A, and I237T variants, the average protein yield was 1–1.5 mg from 1 L of cell pellet. In contrast, all other variants yielded 2–3 mg protein per 1 L of cell pellet.

### Protein expression conditions for the reductase protein VanB

The methods for purifying VanB were previously described and were followed here^7^. First, a single colony of BL21(DE3) *E. coli* that contained the pMCSG7-*vanB* plasmid was used to inoculate a 5 mL starter culture of LB containing 50 µg/mL ampicillin. Second, following overnight incubation at 37 °C, the starter culture was used to inoculate 1 L of LB containing 100 µg/mL ampicillin. These larger cultures were incubated at 37 °C until the OD_600_ reached 0.6–0.8. At this point, flasks were cooled at 20 °C for 1 h prior to the addition of 0.2 mM IPTG. Following an 18 h period of incubation at 20 °C, the cells were harvested, and the protein was purified as described below.

### Purification protocol for the reductase protein VanB

The cell pellet from a 2 L culture of VanB was resuspended in 50 mL of VanB Buffer A (50 mM Tris-HCl pH 8.0, 200 mM NaCl, 20 mM imidazole, 10% glycerol). The mixtures were lysed by sonication using the program described above for SxtT and GxtA. Lysed cells were centrifuged in an Eppendorf centrifuge 5810 R at 12,000 × g for 40 min. The supernatant was loaded onto a Bio-Rad FPLC system fitted with a 5 mL HisTrap HP column (Cytiva). The column was equilibrated using where VanB Buffer A (50 mM Tris-HCl pH 8.0, 200 mM NaCl, 20 mM imidazole, 10% glycerol) and the protein was eluted using VanB Buffer B (50 mM Tris-HCl pH 8.0, 200 mM NaCl, 200 mM imidazole, 10% glycerol). The VanB-containing fractions were pooled and exchanged into storage VanB Buffer C (50 mM HEPES pH 8.0, 100 mM NaCl, 10% glycerol) using a PD-10 desalting column (BioRad). Finally, VanB was concentrated to approximately 200 μM and flash-frozen by liquid nitrogen for long-term storage at −80 °C.

### Circular dichroism (CD) experiments

Stock solutions of wild-type SxtT and SxtT variants stored at concentrations of 250 µM were diluted to 5 µM using a buffer containing 50 mM HEPES pH = 8.0 and 150 mM NaCl. For each CD measurement, 350 µL of the diluted wild-type SxtT or SxtT variant solution was transferred into a 10 mm quartz cuvette (Hellma). The CD spectra were recorded using a Jasco J-1500 CD spectrometer with 0.1 nm data pitch and 20 nm/min scan speeds. The baseline was measured with the same buffer used to do dilution (50 mM HEPES pH = 8.0 and 150 mM NaCl). Each sample spectrum is an average of 5 cumulative spectra.

### Crystallization of β-STOH-bound SxtT, β-STOH-bound GxtA, and STX-bound GxtA

Conditions for crystallizing wild-type and substrate-analog bound SxtT and GxtA were previously identified by our laboratory^7^. To produce crystals of SxtT with its native substrate β-STOH bound, 1 μL of 10 mg/mL SxtT (50 mM HEPES, pH 8.0, 10% v/v glycerol) was combined with 1 μL of well solution of (2.0 M (NH_4_)_2_SO_4_, 0.1 M Bis–Tris pH 6.5, 10% v/v glycerol and 20 mM of β-STOH). In a hanging drop setup, brown crystals appeared within two days of being stored in a 20 °C incubator housed within a Coy chamber (Coy Lab Products) that is maintained using a 95% nitrogen and 5% hydrogen mixture. Likewise, crystals of β-STOH-bound GxtA and STX-bound GxtA were grown by mixing 1 μL of 15 mg/mL GxtA (50 mM HEPES, pH 8.0, 10% v/v glycerol) with 1 μL of well solution (0.3 M MgCl_2_, 0.1 M Bis–Tris pH 5.5, 25% v/v PEG3350, 15% v/v glycerol, and either 20 mM β-STOH or 20 mM STX). Brown crystals appeared within one day following anaerobic incubation at 20 °C. All crystals were harvested and cryocooled in liquid nitrogen without the addition of cryoprotectant in the Coy chamber.

### Xe pressurization experiment with GxtA

The conditions for crystallizing wild-type and substrate-analog bound SxtT and GxtA were previously identified by our laboratory and are described above^7^. However, for this Xe-pressurized structure, we deviated from the conditions described above. To grow crystals for Xe-pressurization experiments, the reservoir solutions did not contain STX or β-STOH and the sitting drop plates used were MiTeGen In-Situ-1 Crystallization Plates. Once brown crystals were identifiable for SxtT and GxtA, the MiTeGen In-Situ-1 Crystallization Plates were shipped to Stanford Synchrotron Radiation Lightsource (SSRL) for Xe incorporation and data collection. Once at SSRL, the crystallization drops were harvested under Paratone N oil using Mitegen loops. Any water-based liquid around any crystals and any extra oil were carefully removed during the harvesting process. These minimal oil-covered crystals were placed inside the SSRL pressurizing apparatus^49^. The GxtA and SxtT crystals were pressurized to 400 psi of Xe gas for 3 min. A subsequent depressurizing period of 15 s was used to return to atmospheric pressure. This step was followed by plunging the pin that contained the looped crystal into a liquid nitrogen container. The frozen Xe-derivatized crystal was transferred and diffraction was measured on an EIGER 16 M detector at SSRL beamline BL14-1. The beam size was adjusted to 100 by 80 microns to match the crystal shape. A total of 4320 images with 0.5 degrees oscillation were collected with inverse beam protocol (every 30 degrees of data were collected close in time to the 180 + 30 degrees of data) to maximize the anomalous signal. For GxtA, three datasets were collected at 6000 eV (2.06633 Å), where the Xe anomalous signal is the strongest. This method resulted in a crystal structure of GxtA that has one atom of Xe in each protomer. These Xe atoms are modeled at an occupancy of 0.8 and their correct placement was confirmed by the calculation of anomalous maps. In contrast to GxtA, the Xe-pressurized SxtT crystals lost diffraction with increased Xe pressures and were not amenable to this experiment.

### Data processing and structure solution

The datasets for β-STOH-bound SxtT, β-STOH-bound GxtA, and STX-bound GxtA were collected at SSRL beamline 9-2 (detector: Dectris Pilatus 6 M). The Xe-GxtA dataset was collected at SSRL beamline 14-1 (described above). For each of the former datasets, the temperature of data collection was 100 K and the wavelength used for data collection was 0.97946 Å (SxtT + β-STOH, GxtA + β-STOH, and GxtA + STX). All datasets were indexed, integrated, and scaled in XDS^50,51^. Similar to that observed for the previously determined structures^7^ of SxtT and GxtA, SxtT indexed as *C*222 and GxtA indexed as *P1*2_1_1. Edited versions of these previously determined structures, which lacked water molecules, metal centers, and small molecules, were used to determine the new β-STOH-SxtT, β-STOH-GxtA, STX-GxtA, and Xe-pressurized structures. Once solved, each model was subjected to five cycles of simulated annealing in Phenix^52^. Subsequent rounds of manual building and adjusting in COOT^53^ coupled with cycles of positional and individual B-factor refinement in Phenix^52^ were used until each model was complete. In general, for each of the structures, protein sidechains were adjusted first, metal centers and water molecules were added second, and ligands were added in the final refinement stages. For the structures of β-STOH-SxtT, β-STOH-GxtA, and STX-GxtA, small molecule parameter files previously^7^ generated using the Grade Web Server Global Phasing (Cambridge, UK) for STX analogs, were also included in refinement. Whereas, for the β-STOH bound GxtA structure, the occupancy of the ligand is set at 1 in each chain of the structure, for β-STOH bound-SxtT and STX-bound GxtA, we see lower occupancy in some of the chains (β-STOH bound-SxtT occupancy = 0.8, 0.6, 0.8 and STX-bound GxtA occupancy = 1, 0.8, 1 for chains A, B, and C, respectively). As the disordered loop in chain A of SxtT with STX bound packs against the same disordered loop of a symmetry mate leaving substantial positive difference electron density at the interface, but no clear way of building the loops without intersection, we re-evaluated the space groups using Pointless^54^ and XTriage in Phenix^52^. We also evaluated other space groups for SxtT, including *C121, C222*_*1*_, *P12*, and *P1*2_1_. However, none of these space groups, other than *C121* allowed us to build a model of SxtT. The resultant lower symmetry model, similar to that observed in the C222 space group also placed the disordered loops from two adjacent protomers close together, confirming our original choice of *C222*. The R_free_ test sets composed of five percent of the original datasets^7^ were used to evaluate the progression of refinement.

After the final refinement stages were completed, the structures were inspected using simulated annealing composite omit electron density maps calculated in Phenix^52^ and the MolProbity program^55^. Ramachandran statistics and omitted residues for each structure determined in this work can be found in Supplementary Table 2. The data collection and refinement statistics for each of the structures are summarized in Supplementary Table 1. Each of the structure figures presented in this work was made using PyMol. The hydropathy plots of SxtT and GxtA were produced in PyMol using the Eisenberg hydrophobicity scale^56^. The crystallography software packages used to determine these structures were compiled by SBGrid^57^.

### Tunnel calculations for proteins studied in this work

The substrate-channeling tunnels in SxtT and GxtA were analyzed as previously described^7^ using the MOLEonline server^43,44^. For tunnel calculation, each of the Rieske oxygenases was loaded into Pymol and exported as monomeric files that contained only the carbon-chain and non-heme iron sites. Additional cofactors if present were removed. Each monomeric subunit was then loaded into the MOLEonline server^43,44^. This analysis was performed for SxtT, GxtA, NdmA (6ICK)^8^, NdmB (6ICL)^8^, Stc2 (3VCP)^12^, KshA (4QCK)^10^, CntA (6Y9D)^11^, OMO (1Z03)^13^, DdmC (3GKE)^9^, and CARDO (2DE7)^14,45^.

### SxtT and GxtA wild-type and variants reactions

Enzymatic reactions were prepared and undertaken following the literature procedure^7^. Wild-type SxtT, SxtT variants, and GxtA were purified as described above and stored in final concentrations of 10 mg/mL. For each enzymatic reaction, a fresh protein aliquot was removed from the freezer. The reactions consisted of either 50 µM wild-type SxtT, SxtT variant, or GxtA. In addition, these reactions contained 36 µM VanB, 200 µM substrate, 500 µM NADH, 100 µM Fe(NH_4_)_2_(SO_4_)_2_, and 50 mM Tris-HCl pH 7.0. Once combined, the reactions were mixed and incubated at 30 °C for 2 h. Reactions were then quenched by the addition of 150 µL acetonitrile and centrifuged. Following centrifugation, 100 µL of the supernatant was diluted with 100 µL of acetonitrile solution that also contained 1% of our mass spectrometry internal standard ^15^N-arginine. The activity of the wild-type SxtT and GxtA proteins, as well as the SxtT variant proteins, was determined by analyzing the amount of substrate consumed as previously described^42^. In particular, the decrease of the substrate peak relative to the control was converted into a percentage of substrate conversion. All reactions presented in this work were run in duplicate.

### Ethanol incorporation into reaction products

To evaluate whether hydroxylation occurred at the C11 or C12 position, ethanol incorporation into the reaction product was probed. First, reactions were conducted as described above. However, following the quenching step and the subsequent addition of the internal standard mixture, 50 µL of 100% ethanol was added to a 50 µL aliquot of each reaction mixture. The resulting mixture was incubated at room temperature for 12 h before analysis by LCMS. To determine the extent of ethanol incorporation into the product, we used a previously documented quantification method, which involves evaluating the amount of incorporated-STX and STX products using standard curves^7^. Again, each reaction presented was run in duplicate. For all assays, the hydroxylation percentage was calculated using Excel and the figures were then made using GraphPad Prism.

### LC–MS and MS/MS analysis

In general, methods described in our previous work, which used an Agilent G6545A quadrupole-time of flight (TOF) or an Agilent 6230 TOF mass spectrometer equipped with a dual AJS ESI source and an Agilent 1290 Infinity series diode array detector, autosampler, and binary pump, were followed here^7^. Solvent A = water with 0.1% formic acid. Solvent B = 95% acetonitrile, 5% water and 0.1% formic acid. An Acquity UPLE BEH Amide 1.7 µm, 2.1 × 100 mm hydrophobic interaction liquid chromatography column was used for all separations (Waters). The chromatographic method was typically 18% A 0–5 min at 0.4 mL/min or 15% A 0–7 min at 0.3 mL/min. 1.0 µL injections were made for each sample.

### Reporting summary

Further information on research design is available in the Nature Research Reporting Summary linked to this article.

## Supplementary information

Supplementary Information

Reporting Summary

## Data Availability

Protein coordinates and structure factors have been submitted to the Protein Data Bank under accession codes 7SZH (SxtT with β-STOH bound), 7SZF (GxtA with β-STOH bound), 7SZE (GxtA with STX bound), and 7SZG (Xenon-pressurized GxtA). The source data underlying Figs. 3–5 and Supplementary Fig. 9–10 are provided as a source data file. Other data are available in the Supplementary Information and from the corresponding authors upon reasonable request. Source data are provided with this paper.

## Source data

Source Data
