# Supplementary material for: Design principles for site-selective hydroxylation by a Rieske oxygenase
Source: Nat Commun. 2022 Jan 11;13:255. doi: 10.1038/s41467-021-27822-3 (PMC8752792; doi:10.1038/s41467-021-27822-3)
Supplement: Supplementary file 1 — Supplementary Information [file 41467_2021_27822_MOESM1_ESM.pdf]

Supplementary Information:

Design Principles for Site-Selective Hydroxylation by a Rieske Oxygenase

Jianxin Liu[1], Jiayi Tian[1], Christopher Perry[1,2, 3], April L. Lukowski[2,3,5], Tzanko I Doukov[4], Alison Narayan[1,2,3]*, Jennifer Bridwell-Rabb[1,2,3]*

***Corresponding Authors:** jebridwe@umich.edu and arhardin@umich.edu

[1]Department of Chemistry, University of Michigan, Ann Arbor, Michigan, 48109
[2]Program in Chemical Biology, University of Michigan, Ann Arbor, Michigan, 48109
[3]Life Sciences Institute, University of Michigan, Ann Arbor, Michigan, 48109
[4]Macromolecular Crystallography Group, Stanford Synchrotron Radiation Light Source, SLAC National Accelerator Laboratory, Menlo Park, CA 94025
[5]Present Address: Center for Marine Biotechnology and Biomedicine, Scripps Institution of Oceanography, University of California, San Diego, La Jolla, CA, 92037

```
                 --α_a--   --β1--α1-   ---β2---   ---β3---      --β4--
SxtT      MTTADLILINNWHVVANVEDCKPGSITTARLLGVKLVLWRSQEQNSPIQVWQDYCPHRGV      60
GxtA      MTTADLILINNWYVVAKVEDCRPGSITTAHLLGVKLVLWRSHEQNSPIQVWQDYCPHRGV      60
          ************:***:****:*******:***********:******************

           -α_b--β5- -β6-   -β7-    -β8-                   --β9--  -β10-
SxtT      PLSMGEVANNTLVCPYHGWRYNQAGKCVQIPAHPDMVPPASAQAKTYHCQERYGLVWVCL      120
GxtA      PLSMGEVANNTLVCPYHGWRYNQAGKCVQIPAHPDMVPPASAQAKTYHCQERYGLVWVCL      120
          ************************************************************

                    -α_c-    -β11_a- -β11_b-  --α2--     --α3--
SxtT      GNPVNDIPSFPEWDDPNYHKTYTKSYLIQASPFRVMDNSIDVSHFPFIHDGWLGDRNYTK      180
GxtA      GNPVNDIPSFPEWDDPNYHKTYTKSYLIQASPFRVMDNSIDVSHFPFIHEGILGDRNHAE      180
          *************************************************:* *****:::

              -β12-    ---β13---            ---β14---  ---β15-   --β16-
SxtT      VEDFEVKVDKDGLTMGKYQFQTSRIVSHIEDDSWVNWFRLSHPLCQYCVSESPEMRIVDL      240
GxtA      VEDLEVKVDKDGLTMGKYQVHTSKFNNSTKDDSMVNWFRLSHPLCQYCSTEASEMRTVDL      240
          ***:***************.:**:: .  :*** ************** :*: *** ***

           ------    ----β17---     ----------α4-----------
SxtT      MTIAPIDEDNSVLRMLIMWNGSEMLESKMLTEYDETIEQDIRILHSQQPARLPLLAPKQI      300
GxtA      MVVTPIDEDNSVLRYLIMWNGSKTLESKILADYDQVIEEDIRILHSQQPTRLPLLSPKQI      300
          *.::********** *******: ****:*::**:.**:**********:*****:****

                   -α5_a- -----α5_b-----
SxtT      NTQGLPQEIHVPSDRGTVAYRRWLKELGVTYGVC      334
GxtA      NTQGLPQEIHVPSDRCTVAYRRWLKELGVTYGVC      334
          *************** ******************
```

Supplementary Figure 1. SxtT and GxtA share high levels of sequence identity with one another. The secondary structure observed in the crystal structures of SxtT and GxtA are marked above the alignment. The α-helices marked a, b, and c are not universally conserved among structurally characterized Rieske oxygenases[1]. The metal-binding residues are marked in green. The flexible loop region is highlighted in grey. Arg204 from the loop region is shown in blue, whereas tunnel lining residues identified in this work as being important for selectivity are shown in purple. Active site residues that are important for selectivity are shown in red

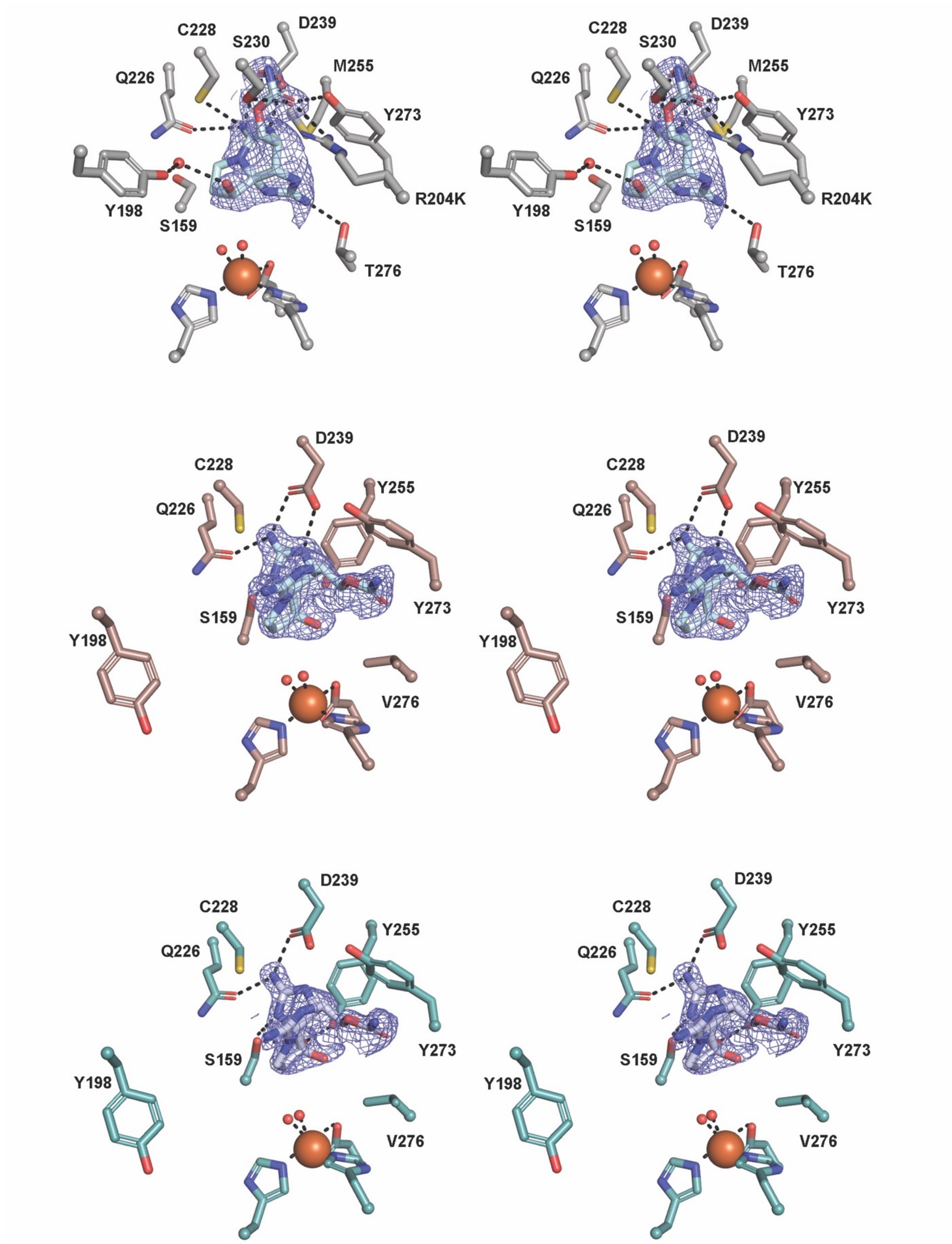

Supplementary Figure 2. Stereo figures of β-STOH bound to SxtT (top), GxtA (middle), and STX bound to GxtA (bottom). All panels are shown with 2Fo-Fc simulated annealing composite omit electron density maps. These maps are contoured at 0.9σ for SxtT and 1.0σ for GxtA.

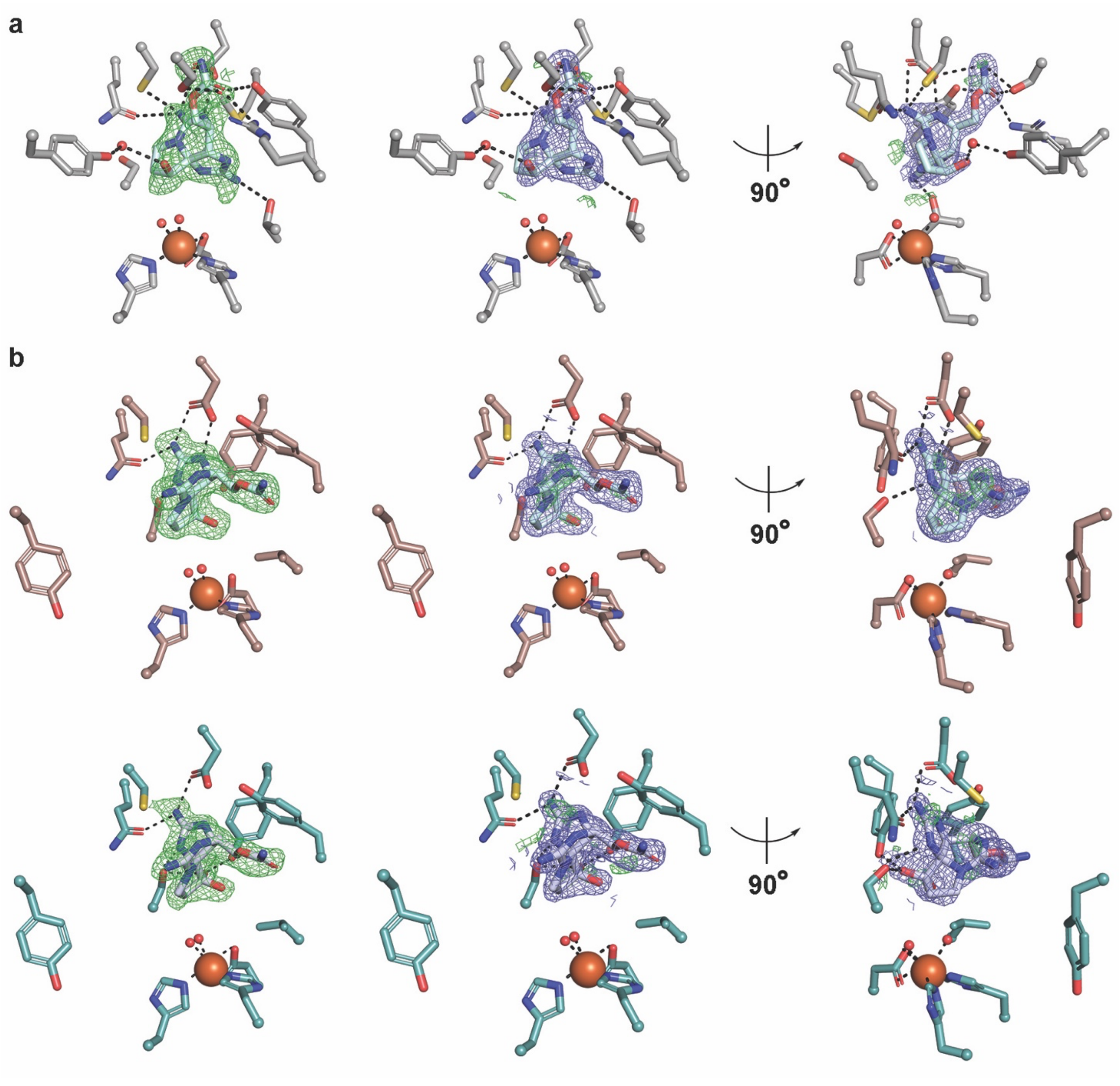

Supplementary Figure 3. β-STOH and STX bind in remarkably different orientations in SxtT and GxtA. (a) In the left panel, a Fo-Fc omit electron density map contoured at ±3.0σ was calculated after β-STOH was omitted from the refined structure of β-STOH-bound SxtT. In the right two panels, two orientations of β-STOH bound to SxtT are shown with 2Fo-Fc and Fo-Fc electron density maps contoured at 1.0σ and ±3.0σ, respectively. (b) Similar to panel a, a Fo-Fc omit electron density map contoured at ±3.0σ around β-STOH bound to GxtA as well as refined 2Fo-Fc and Fo-Fc electron density maps contoured at 1.0σ and ±3.0σ, respectively, are shown. (c) STX makes extra interactions in the GxtA active site. STX is shown in the left panel with an omit electron density map contoured at 3.0σ after STX was omitted from the STX-bound structure. The right panels show 2Fo-Fc and Fo-Fc electron density maps contoured at 1.0σ and ±3.0σ, respectively.

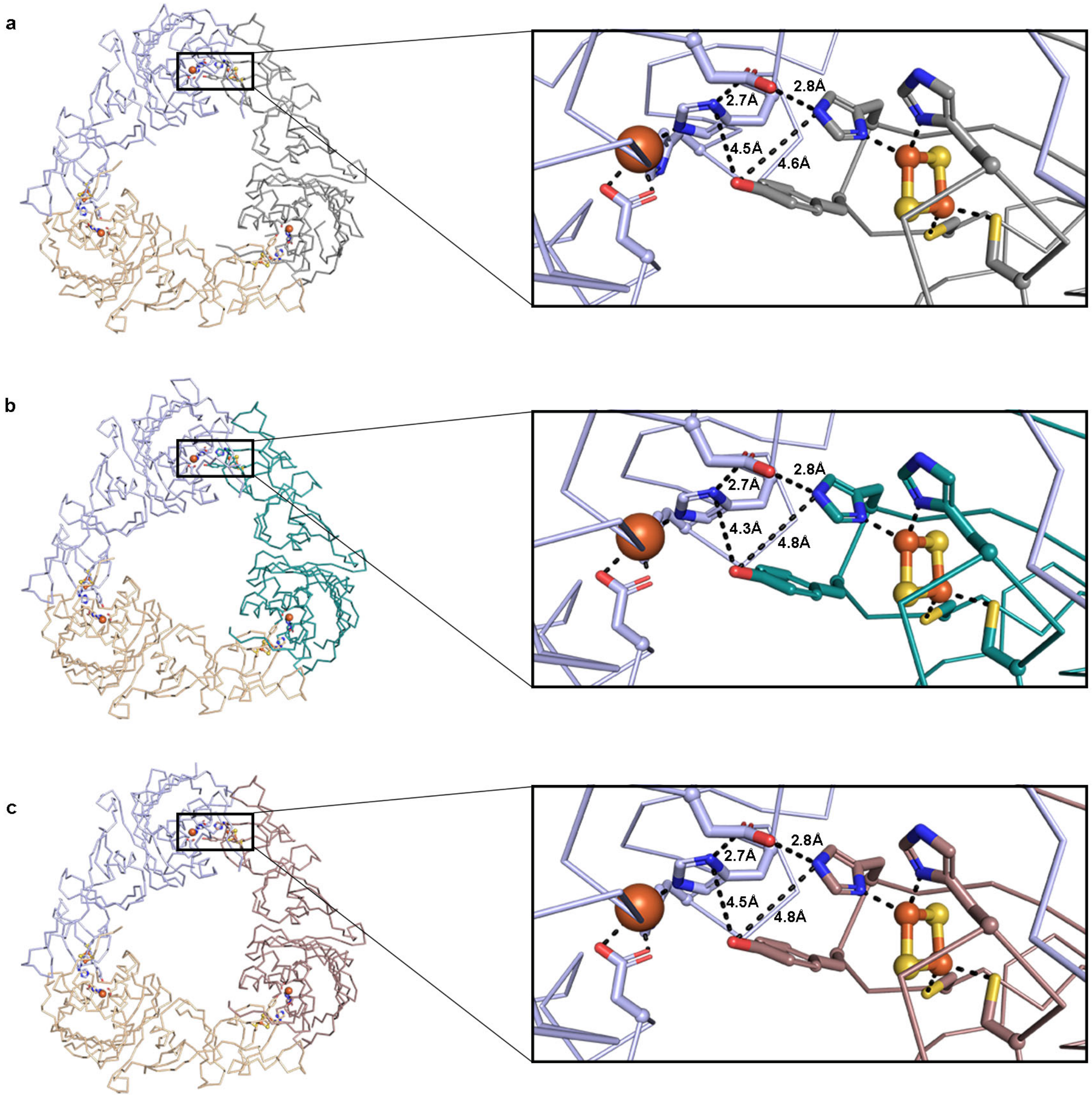

Supplementary Figure 4. The structures of SxtT and GxtA determined in this work each has a trimeric architecture. Each monomeric unit of the contains a [2Fe-2S] Rieske cluster in the N-terminal domain and a mononuclear iron center in the C-terminal domain. As observed in other Rieske oxygenase structures, in all of the substrate bound structures determined here, the metallocenters of two different subunits are placed within electron transfer distance of one another across the subunit-subunit interfaces. The path between the Rieske cluster from one subunit and the mononuclear iron from a second subunit are shown in enlarged panels for the three substrate bound structures determined here. (a) The structure of SxtT with β-STOH bound. (b) The structure of GxtA with STX bound. (c) The structure of GxtA with β-STOH bound.

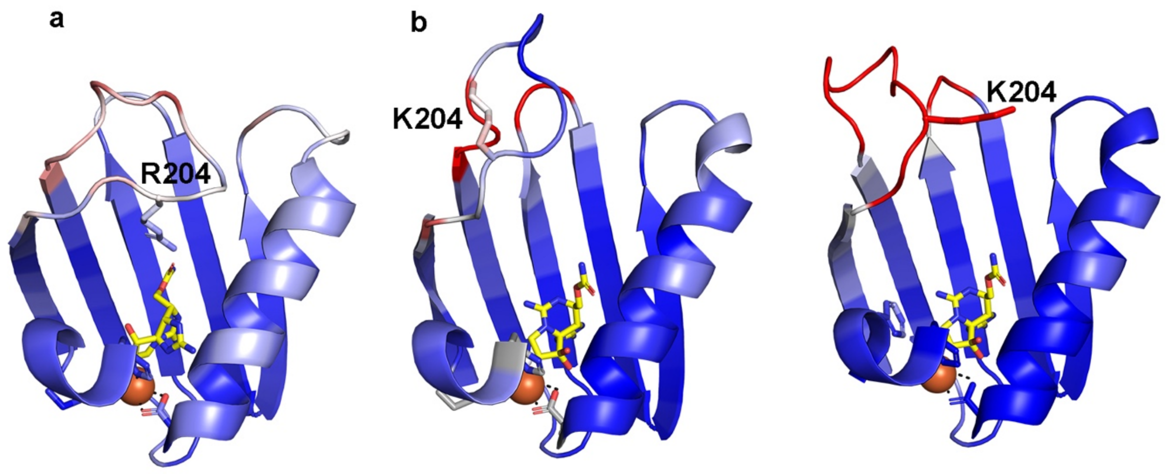

Supplementary Figure 5. Identification of a flexible loop that is important for selectivity. The loop that connects the β13 and β14 strands of the C-terminal iron binding domain in SxtT and GxtA is highly flexible as evidenced by the B-factors. (a) The flexible loop in SxtT reaches toward the active site and Arg204 interacts with β-STOH. (b) The two orientations of the loop in STX-bound GxtA extend away from the active site in both the β-STOH and STX-bound structures. In all panels, the B-factors are plotted on a scale of 20–60 $Å^2$ with increasing values colored in blue, white, and red, respectively.

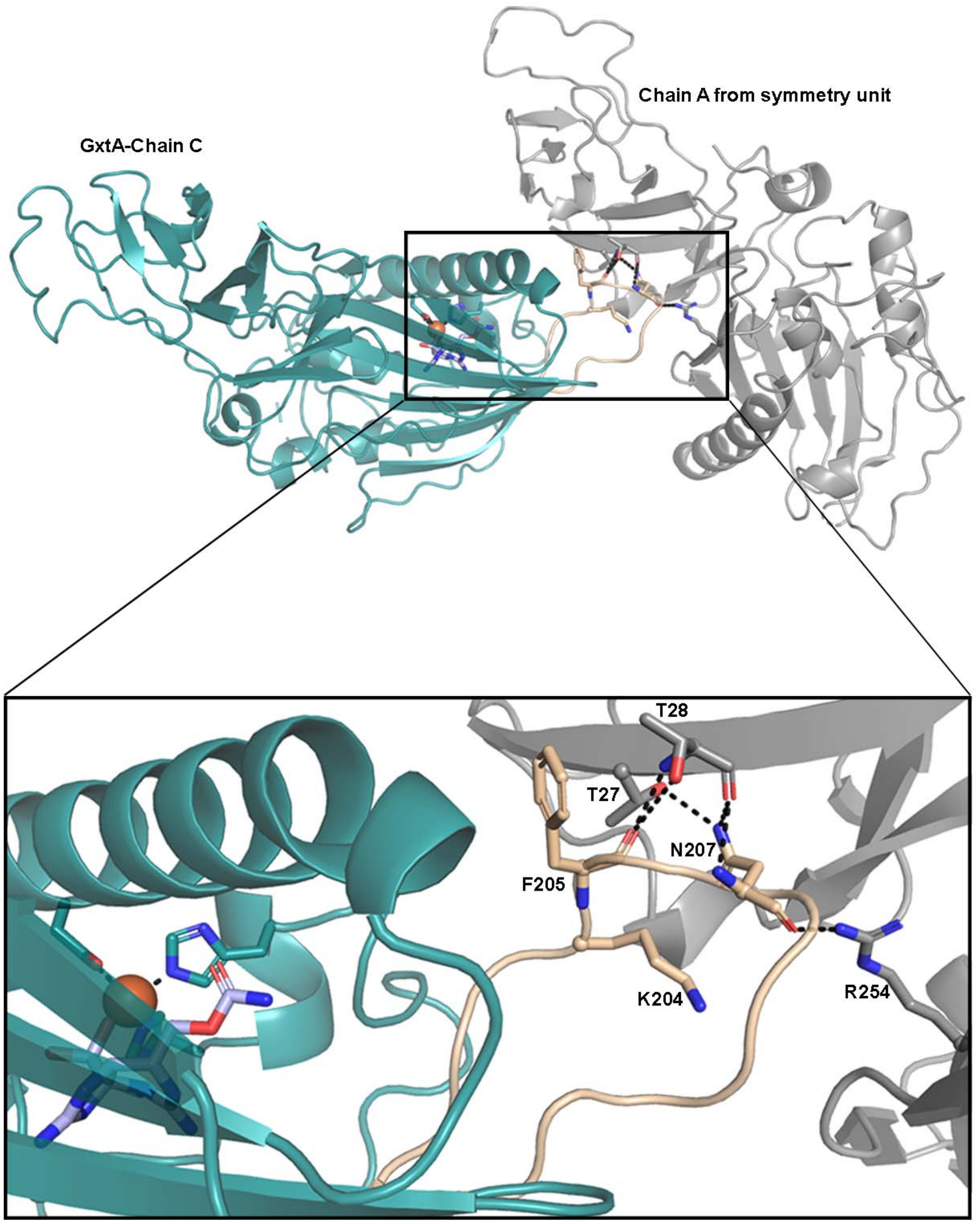

Supplementary Figure 6. The structure of GxtA shows two orientations of the flexible loop. These orientations can be seen in Figure 3b. One of the loop orientations is extended (wheat) and stabilized by interactions with a symmetry related molecule in the crystal packing. This extended loop is shown here in wheat as part of the dark teal monomer. The symmetry related molecule that is interacting with this loop is shown in gray. A zoomed in view of the interactions made between the loop and the symmetry molecule are shown in the bottom panel.

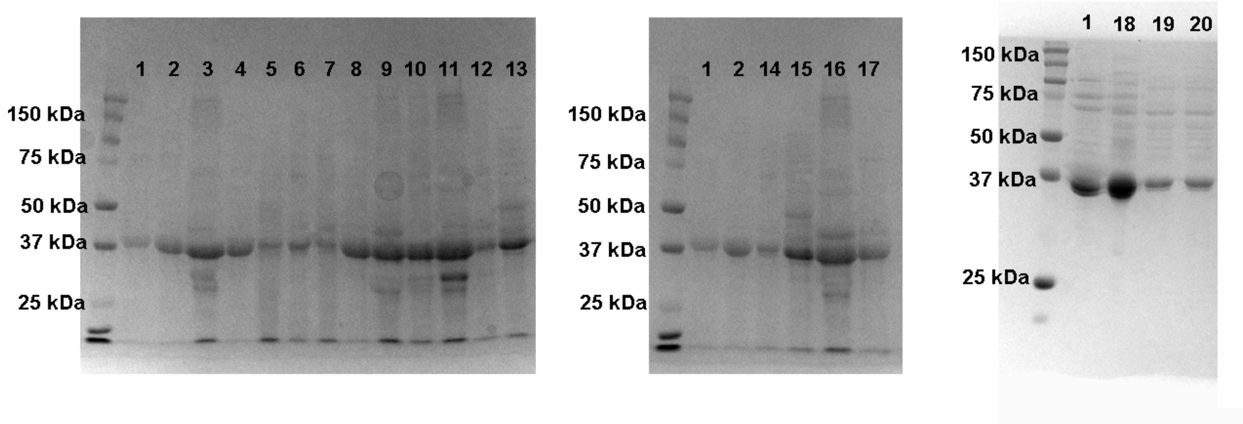

1. SxtT
2. GxtA
3. M255Y/T276V/R204K
4. W214M/S232A/I237T (tunnel chimera)
5. M255Y/T276V/R204K/S232A
6. M255Y/T276V/R204K/I237T
7. M255Y/T276V/R204K/W214M
8. M255Y/T276V/R204K/W214M/S232A/I237T
9. M255Y/T276V/W214M/S232A/I237T
10. S232A
11. I237T
12. S232A/I237T
13. W214M/I237T
14. F200V/Q201H/R204K/I205F/V206N/S207N/H208S/I209T/E210K/W214M (loop chimera)
15. M255Y/T276V/R204K
16. M255Y/T276V
17. R204K
18. F200V/Q201H/R204K
19. F200V/Q201H/R204K/I205F/V206N/S207N
20. F200V/Q201H/R204K/I205F/V206N/S207N/H208S/I209T/E210K

Supplementary Figure 7. The wild type SxtT and GxtA proteins as well as the SxtT variants used in this work were expressed and purified using affinity and gel filtration chromatography. The purity of each protein was assessed using SDS-PAGE gels. As predicted, each wild-type and variant protein has a molecular weight of approximately 38 kDa (judged based on comparison to a protein standard). Aliquots of these purified proteins were used once and discarded to ensure that consistent activity was observed across the batch.

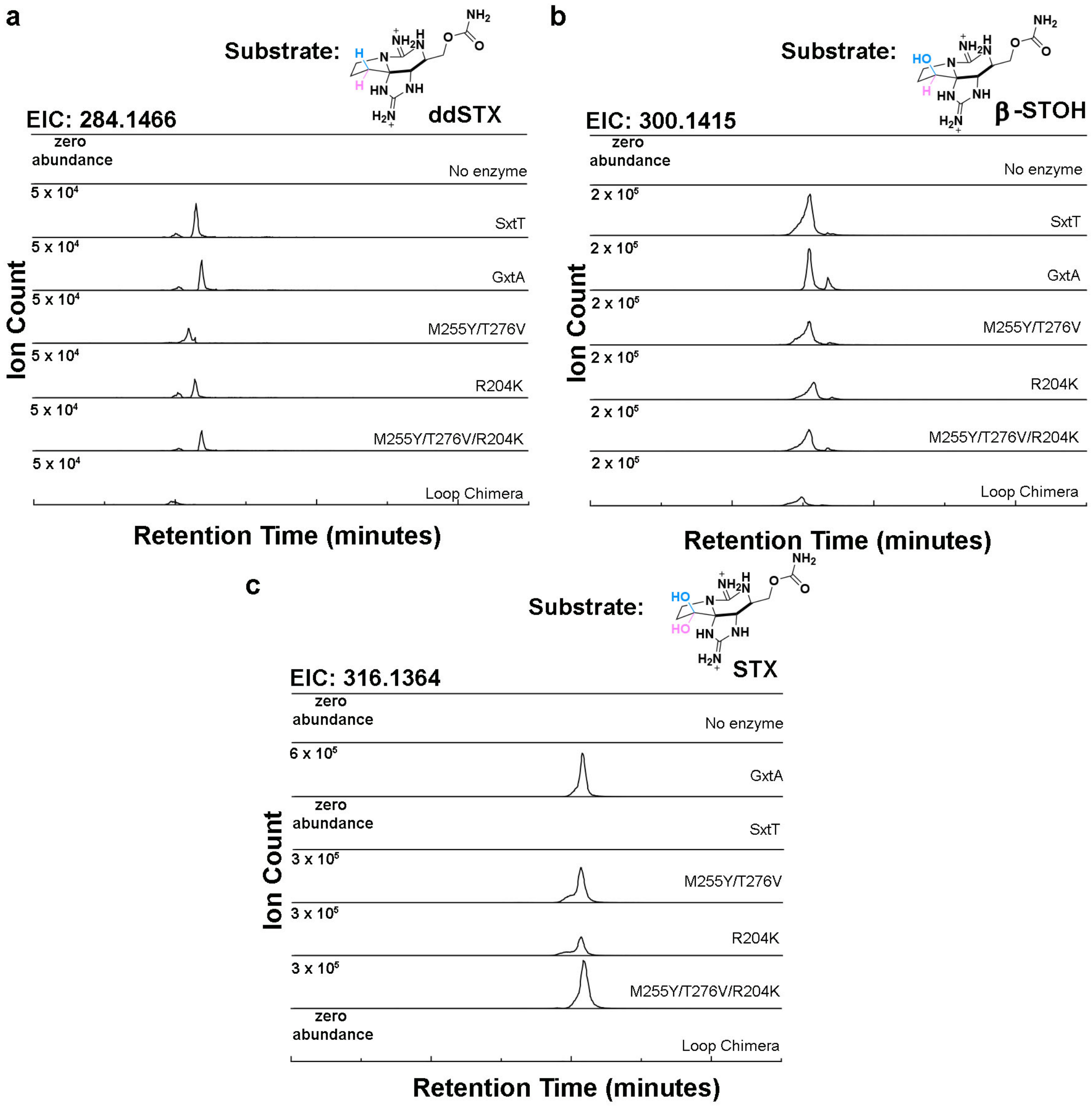

Supplementary Figure 8. A combination of changes to the active site and flexible loop of SxtT leads to an increased ability to hydroxylate STX. (a) The extracted ion chromatograms for SxtT variant reaction products with a ddSTX substrate. The m/z = 284.1466 is the mass of a hydroxylated ddSTX product. (b) The extracted ion chromatograms of the hydroxylated product formed for SxtT variants with a β-STOH substrate (m/z = 300.1415). (c) The extracted ion chromatograms of the hydroxylated product formed for SxtT variants with a STX substrate (m/z = 316.1364).

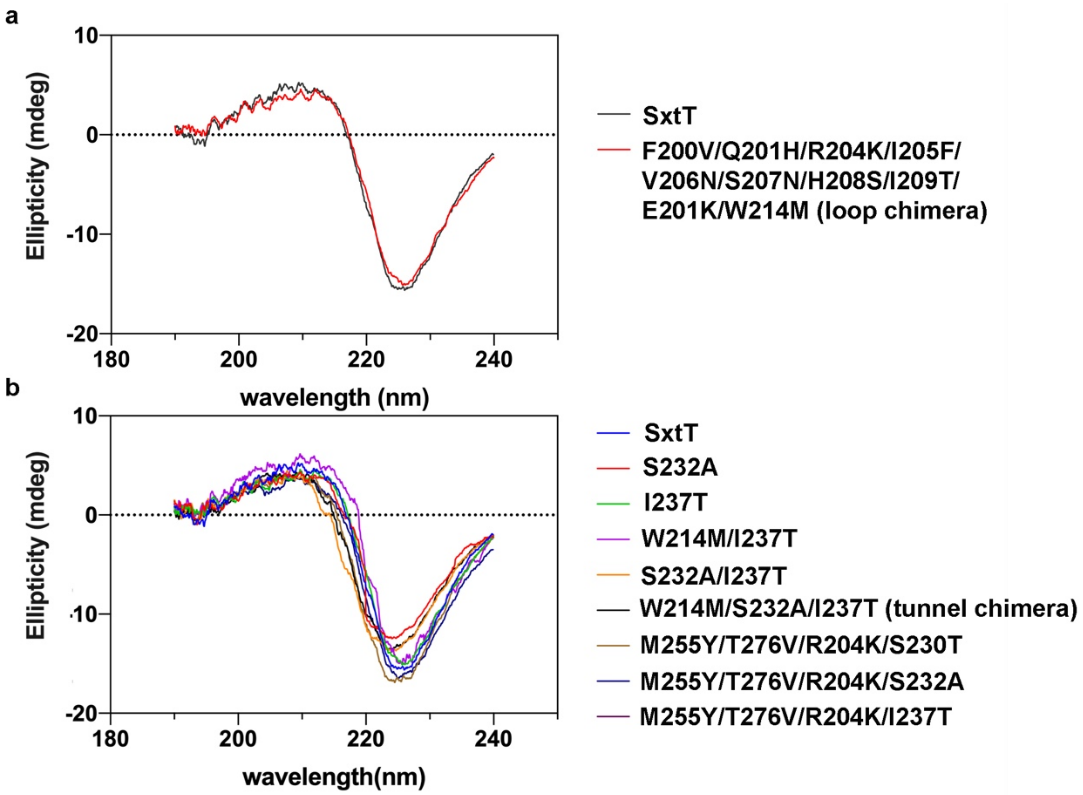

Supplementary Figure 9. Despite low levels of measured activity, variants in the loop and tunnel region of SxtT are folded. (a) Circular dichroism (CD) data shows that the SxtT loop chimera variant (F200V, Q201H, R204K, I205F, V206N, S207N, H208S, I209T, E210K, W214M) resembles wild-type SxtT. (b) Similarly, CD data on single, double and triple variants in the tunnel region resemble wild-type SxtT. This experiment, along with gel filtration chromatography, iron analysis, and SDS-PAGE, was performed with the samples assayed in this work to ensure that the proteins used were pure, homogenous, reconstituted, and folded. Source data are provided as a Source Data file.

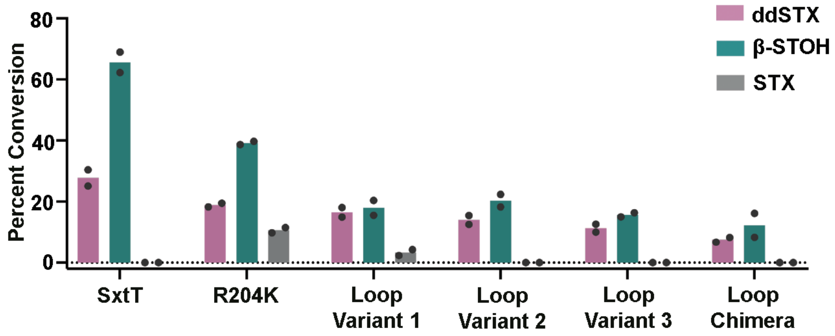

Loop Variant 1: F200V/Q201H/R204K
Loop Variant 2: F200V/Q201H/R204K/I205F/V206N/S207N
Loop Variant 3: F200V/Q201H/R204K/I205F/V206N/S207N/H208S/I209T/E210K
Loop Chimera: F200V/Q201H/R204K/I205F/V206N/S207N/H208S/I209T/E210K/W214M

Supplementary Figure 10. Changes made to the flexible loop of SxtT so that it resembles GxtA leads to a decreased ability to hydroxylate ddSTX and β-STOH. As shown in Fig. 3, an R204K SxtT variant shows activity on each of the three provided substrates whereas a full loop chimera shows reduced activity on ddSTX and β-STOH and no activity on STX. Only one of the shorter loop chimera variants, F200V/Q201H/R204K, shows any activity on STX. Of note, this variant has reduced activity on all three provided substrates relative to wild type SxtT and the R204K variant. Data was measured data was measured using n=2 independent experiments. Source data are provided as a Source Data file.

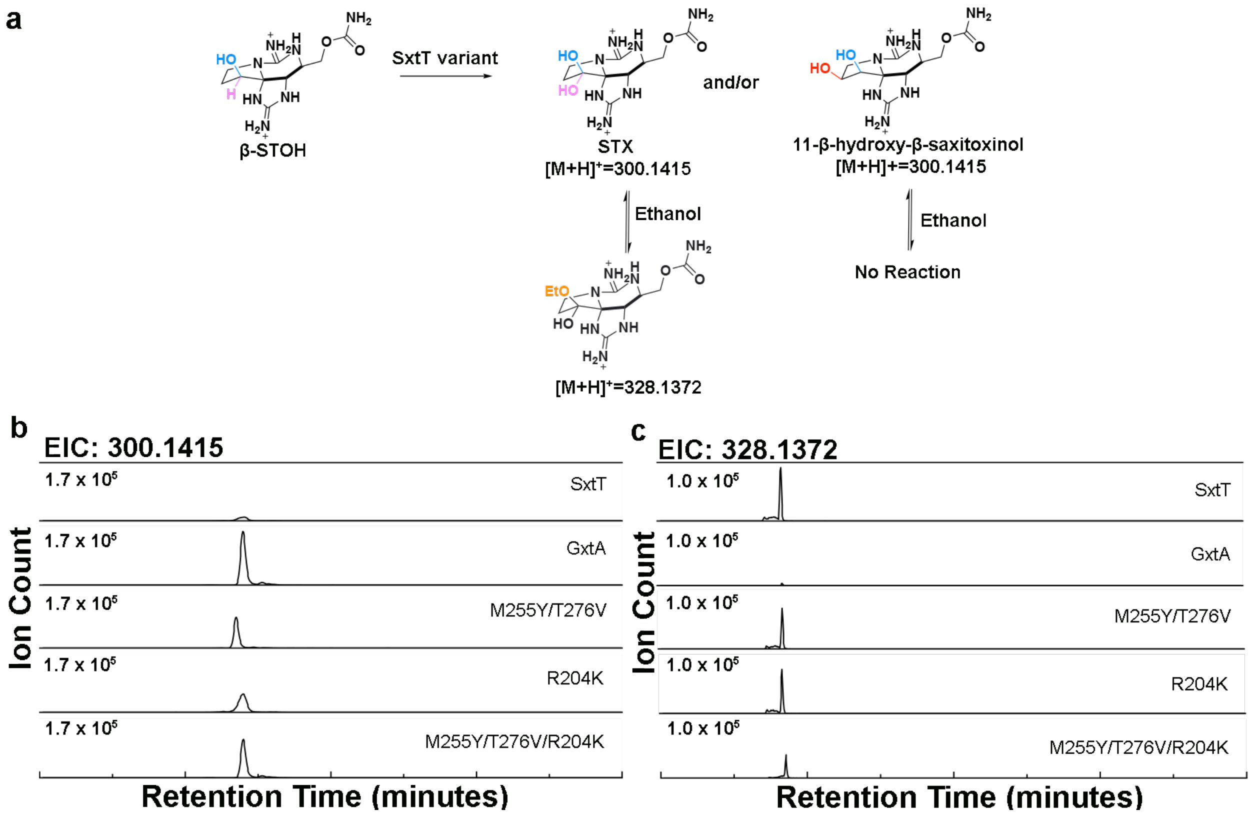

Supplementary Figure 11. Hydroxylation on β-STOH happens at the C11 and C12 positions in SxtT M255Y/T276V, R204K, and M255Y/T276V/R204K variants**.** (a) If the product of β-STOH hydroxylation by a SxtT variant is STX (hydroxylation at the C12 position), it can be monitored by looking for incorporation of ethanol into the product. If the SxtT variant instead catalyzes hydroxylation at the C11 position, ethanol will not be incorporated. (b) Extracted ion chromatograms of the SxtT, GxtA, and M255Y/T276V, R204K, and M255Y/T276V/R204K variant reaction product with of β-STOH. The m/z = 300.1415 value represents the mass of 11-β-hydroxy-β-STOH or STX. (c) Extracted ion chromatograms of the SxtT, GxtA, and M255Y/T276V, R204K, and M255Y/T276V/R204K variant reaction product with β-STOH. The m/z = 328.1372 value represents the mass of an ethanol incorporated product, which is only formed when hydroxylation happens at the C12 position.

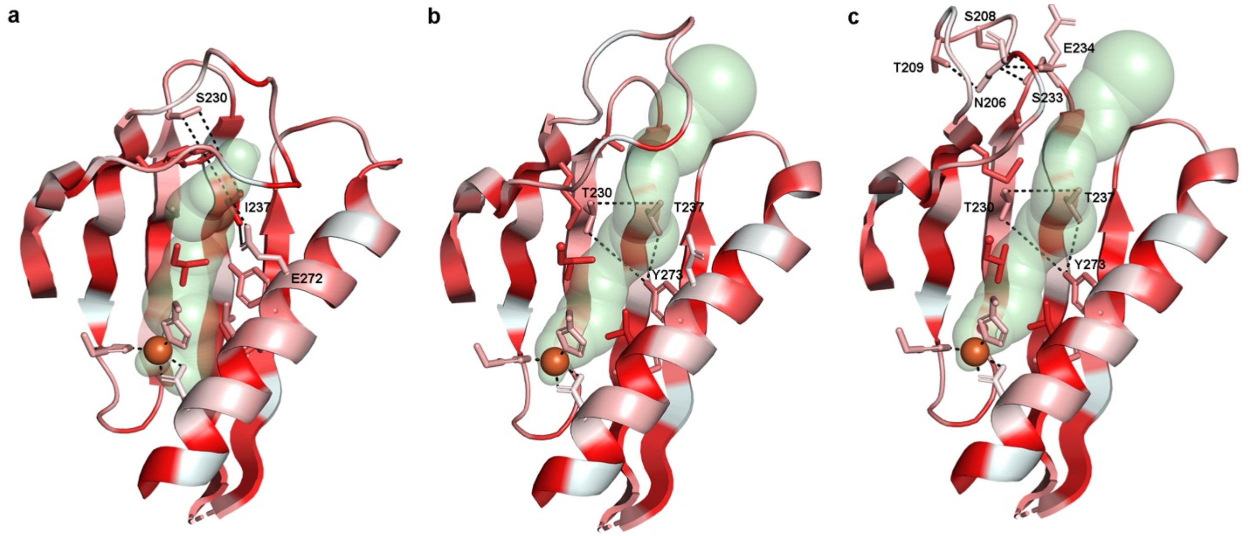

Supplementary Figure 12. Comparison of the tunnels and hydropathy index of SxtT and GxtA. (a) The residues that define the bottleneck of SxtT are labeled and dashed lines are drawn between them. The bottleneck of the tunnel appears to be at the entrance. Of note, due to the closed nature of the loop, the tunnel was calculated without residue Arg204 being present. (b-c) An equivalent portion of GxtA to that detailed for SxtT in panel a is shown with the residues that define the bottleneck labeled and connected by dashed lines. Unlike SxtT, the bottleneck of the GxtA tunnel is centrally located. In addition, the tunnel in GxtA is more hydrophobic than the one in SxtT and the flexible loop is more polar. All panels are colored based on the Eisenberg hydrophobicity scale[2].

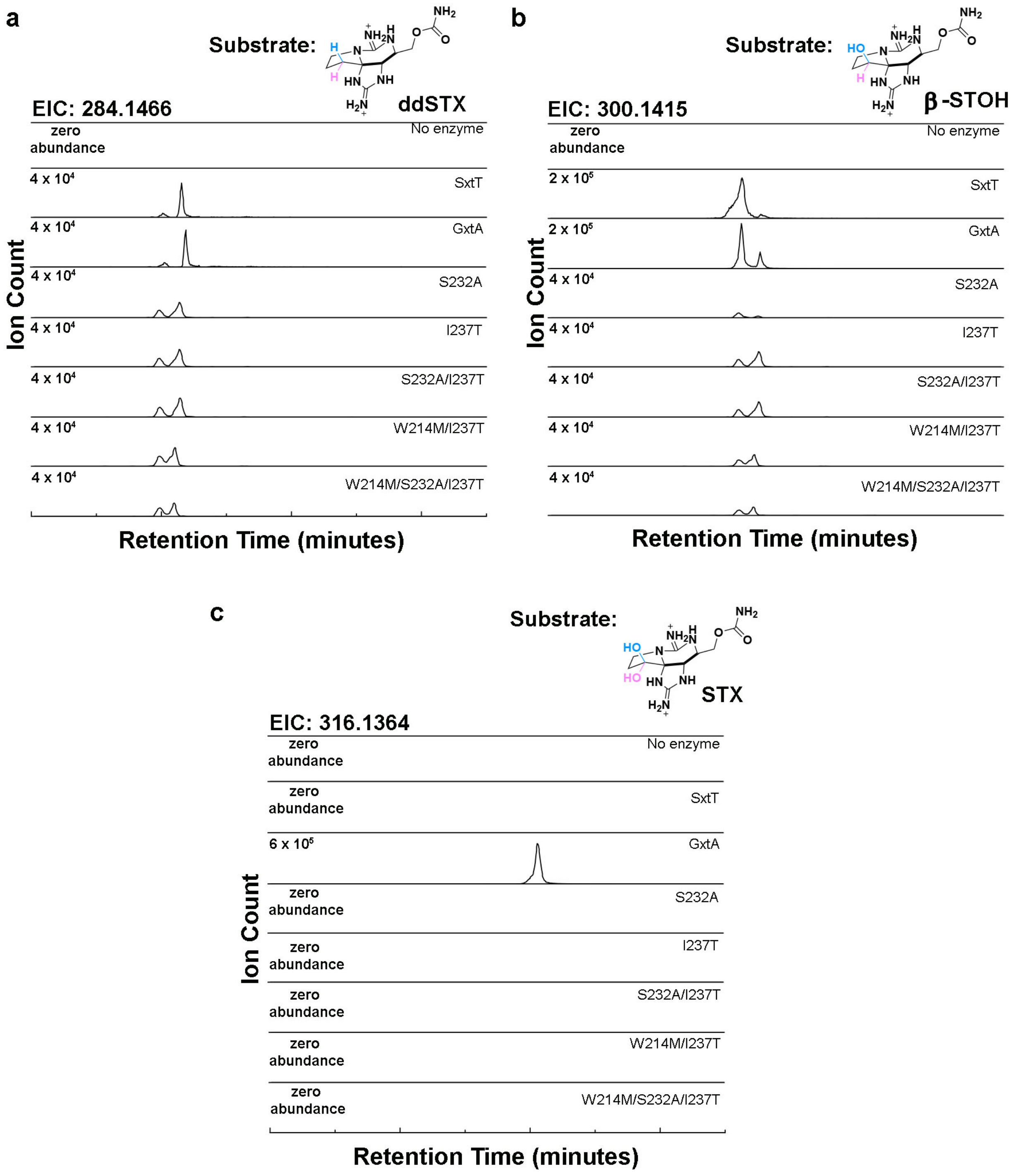

Supplementary Figure 13. Variants of SxtT in the tunnel region show low levels of activity on ddSTX, β-STOH, and no activity on STX. (a) The extracted ion chromatograms for SxtT tunnel variant reaction products with a ddSTX substrate. The m/z = 284.1466 is the mass of a hydroxylated ddSTX product. (b) The extracted ion chromatograms of the hydroxylated product formed for SxtT tunnel variants with a β-STOH substrate (m/z = 300.1415). (c) The extracted ion chromatograms of the hydroxylated product formed for SxtT tunnel variants with a STX substrate (m/z = 316.1364). As shown here, none of the single, double, or tunnel chimera variant have activity on STX.

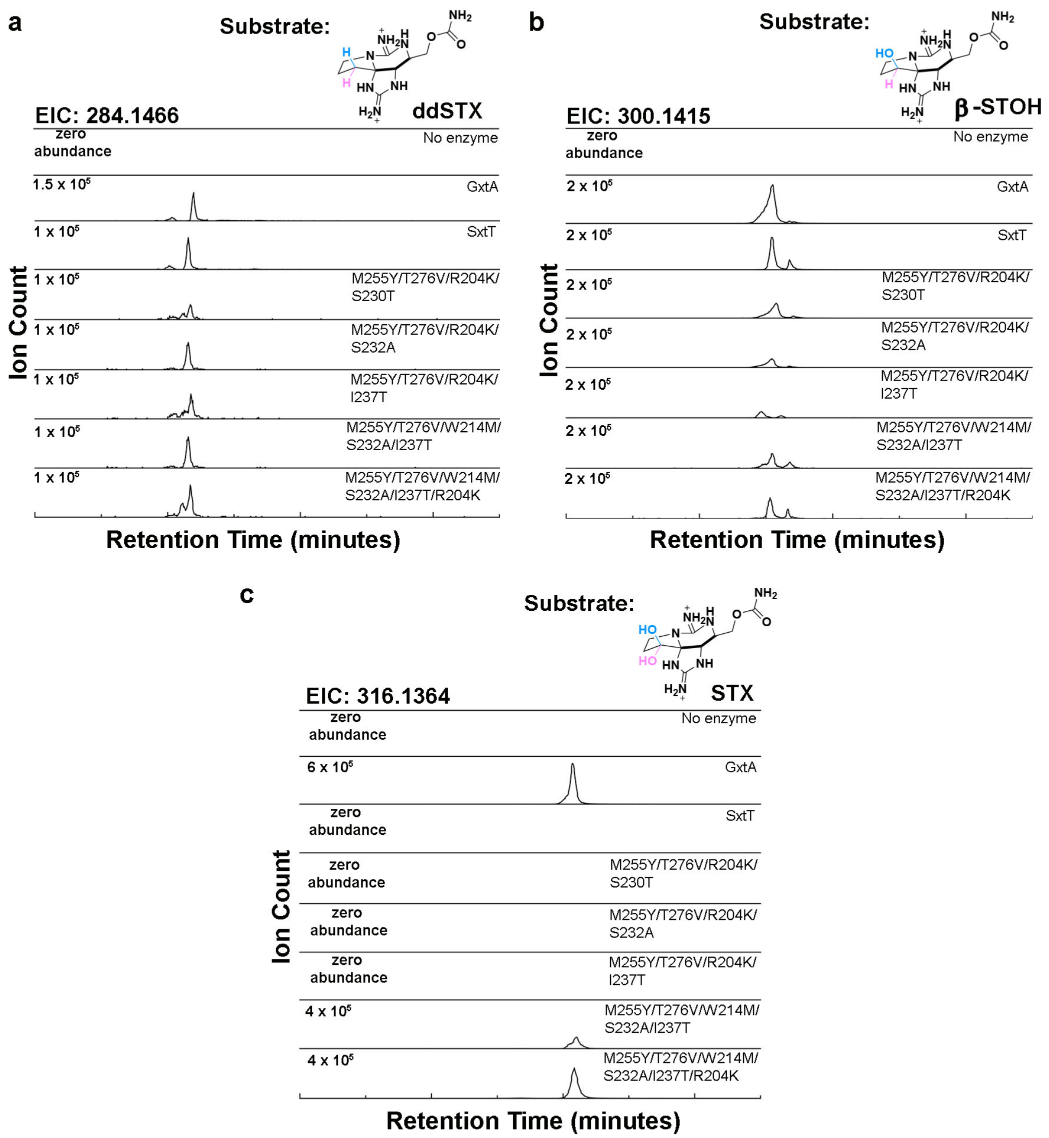

Supplementary Figure 14. Variants of SxtT that combine changes in the tunnel chimera with changes in the active site and loop region show differing levels of activity on ddSTX, β -STOH, and STX. (a) The extracted ion chromatograms for SxtT variant reaction products with a ddSTX substrate. The m/z = 284.1466 is the mass of a hydroxylated ddSTX product. (b) The extracted ion chromatograms of the hydroxylated product formed for SxtT variants with a β-STOH substrate (m/z = 300.1415). (c) The extracted ion chromatograms of the hydroxylated product formed for SxtT variants with a STX substrate (m/z = 316.1364). As shown here, the M255Y/T276V/R204K, M255Y/T276V/W214M/S232A/I237T, and M255Y/T276V/R204K/W214M/S232A/I237T variants can each hydroxylate STX, suggesting they can now hydroxylate at C11.

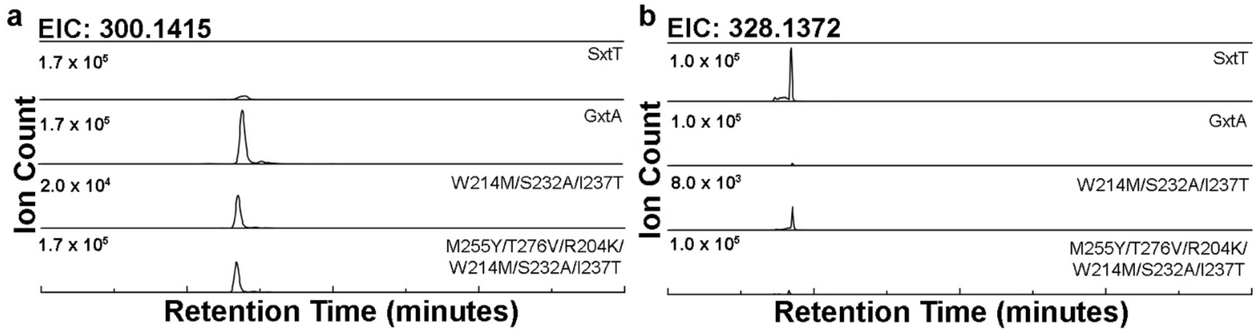

Supplementary Figure 15. A SxtT variant that combines changes in the active site, loop, and tunnel region, mirrors the selectivity of GxtA and catalyzes hydroxylation primarily at C11. (a) Extracted ion chromatograms of the SxtT, GxtA, W214M/S232A/I237T variant, and M255Y/T276V/R204K/W214M/S232A/I237T variant reaction product with of β-STOH. The m/z = 300.1415 value represents the mass of 11-β-hydroxy-β-STOH. (b) Extracted ion chromatograms of the SxtT, GxtA, W214M/S232A/I237T variant, and M255Y/T276V/R204K/W214M/S232A/I237T variant reaction product with β-STOH. The m/z = 328.1372 value represents the mass of an ethanol incorporated product. The reaction scheme for this figure can be seen in Supplementary Figure 11a.

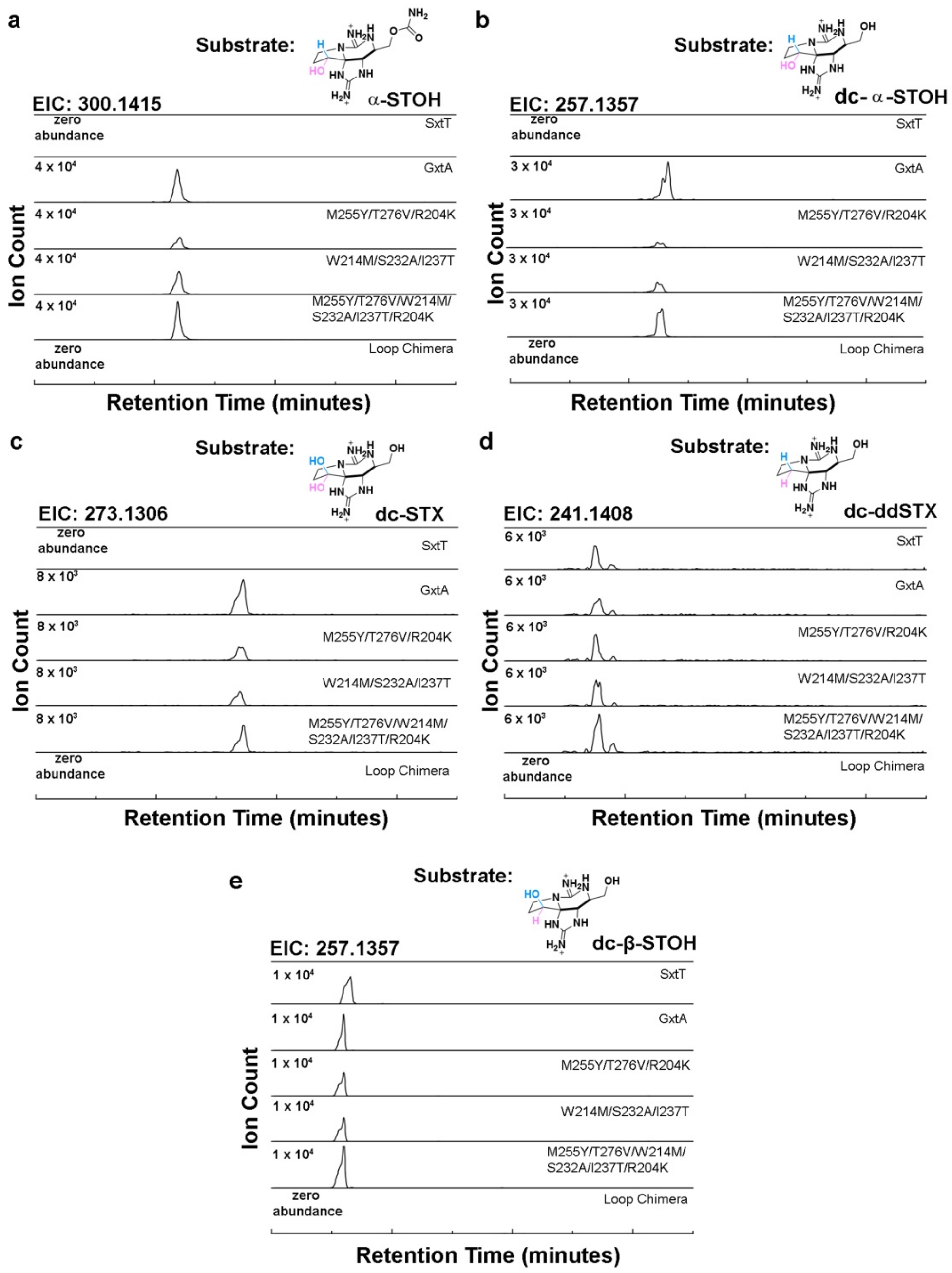

Supplementary Figure 16. Variants of SxtT that combine changes in the active site, tunnel, and loop region show activity on α-STOH, dc-α-STOH, dc-STX, dc-ddSTX and dc-β-STOH. (a) The extracted ion chromatograms for SxtT variant reaction products with an α-STX substrate. The m/z = 300.1415 is the mass of a hydroxylated α-STOH product. (b) The extracted ion chromatograms of the hydroxylated product formed for SxtT variants with a dc-α-STOH substrate (m/z = 257.1357) (c) The extracted ion chromatograms of the hydroxylated product formed for SxtT variants with a dc-STX substrate (m/z = 273.1306) (d) The extracted ion chromatograms of the hydroxylated product formed for SxtT variants with a dc-ddSTX substrate (m/z = 241.1408) (e) The extracted ion chromatograms of the hydroxylated product formed for SxtT variants with a dc-β-STOH substrate (m/z = 257.1357). In these panels, ddSTX corresponds to dideoxysaxitoxin, β/α-STOH corresponds to β/α-saxitoxinol, and STX corresponds to saxitoxin. The abbreviation dc- represents dicarbamoyl.

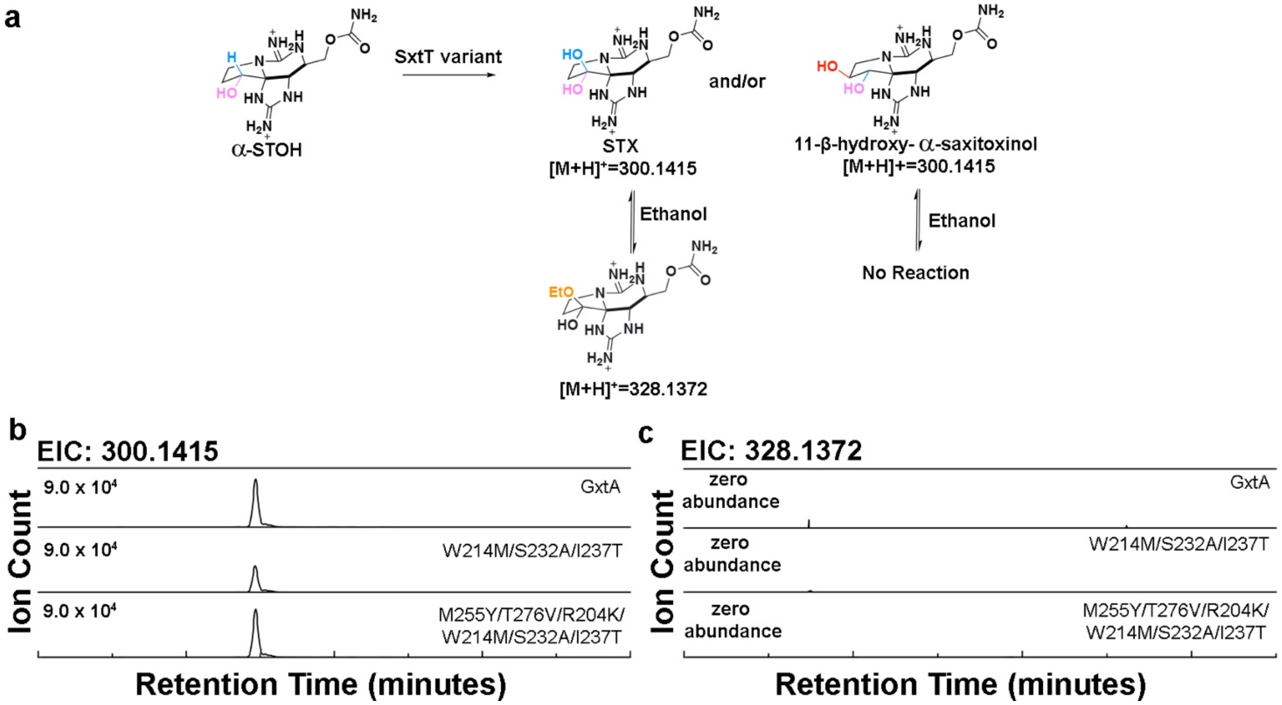

Supplementary Figure 17. The SxtT tunnel chimera variant and variant that combines changes in the active site, loop, and tunnel region catalyzes hydroxylation at the C11 position of α-STOH. (a) Extracted ion chromatograms of the GxtA, W214M/S232A/I237T variant, and M255Y/T276V/R204K/W214M/S232A/I237T variant reaction product with α-STOH. The m/z = 300.1415 value represents the mass of 11-β-hydroxy-α-STOH. (b) Extracted ion chromatograms of the GxtA, W214M/S232A/I237T variant, and M255Y/T276V/R204K/W214M/S232A/I237T variant reaction product with α-STOH. The m/z = 328.1372 value represents the mass of an ethanol incorporated product, which would only be observed if hydroxylation occurred at C12.

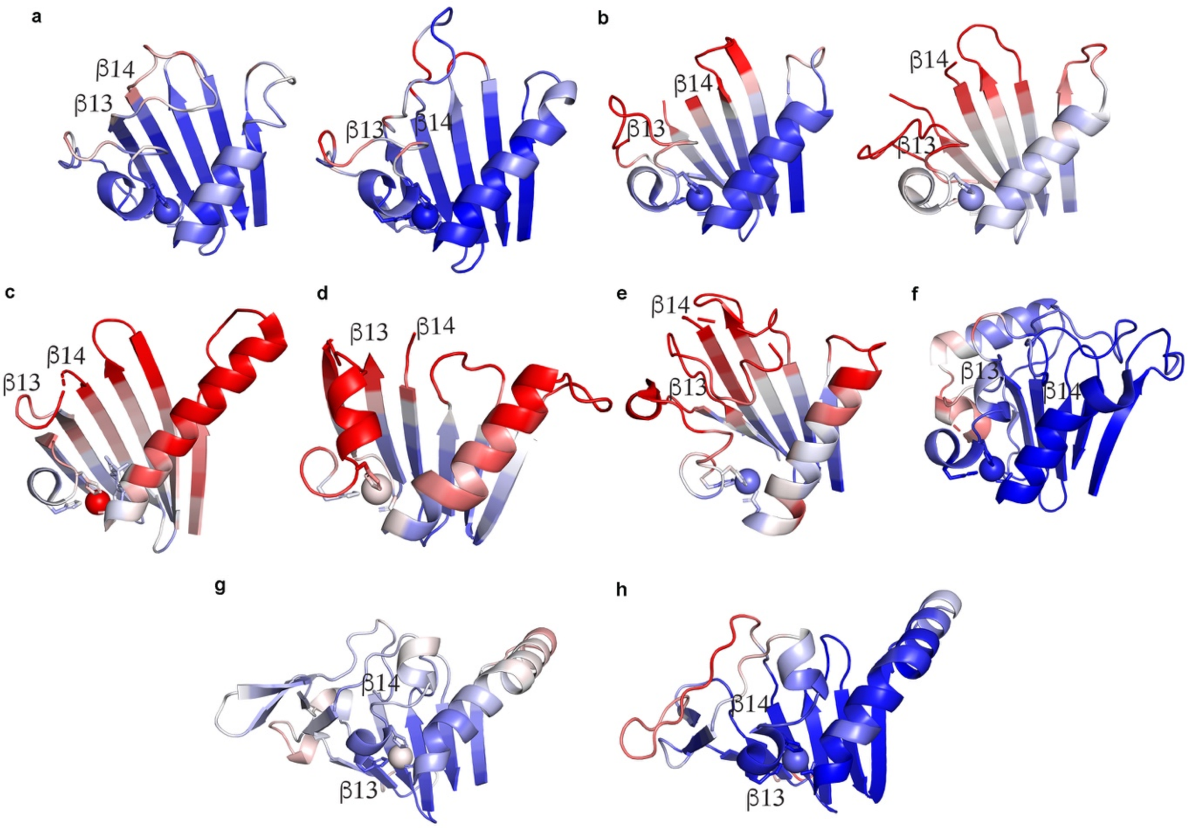

Supplementary Figure 18. The loop that connects the β13 and β14 strands of the C-terminal iron binding domain in SxtT and GxtA, as well as in most other structurally characterized α_3 Rieske oxygenases, is flexible. (a) For reference the structures of SxtT (left) and GxtA (right) are provided for comparison. (b) The loop that connects β13 and β14 in NdmA (left) and NdmB (right) is missing from the crystal structures due to disorder (PDB: 6ICK, 6ICL)[3]. (c) A flexible loop that is partly missing connects β13 and β14 in KshA (PDB: 4QCK)[4]. (d) CntA (PDB: 6Y9D)[5], like NdmA and NdmB, has a β13 and β14 connecting loop that is mostly missing. (e) DdmC (PDB: 3GKE)[6] also has a slight disordered and unmodeled loop that connects β13 and β14 in the C-terminal domain. (f) There is some amount of flexibility in the Stc2 (PDB: 3VCP)[7] loop, but it is longer than the SxtT and GxtA loops. (g) Finally, CARDO (PDB: 2DE7)[8,9] and (h) OMO (1Z03)[10] both showcase very long loops that have extra secondary structure. The loop of OMO is more flexible than that of CARDO. In all panels, the B-factors are plotted on a scale of 20–60 Å^2 with increasing values colored in blue, white, and red, respectively.

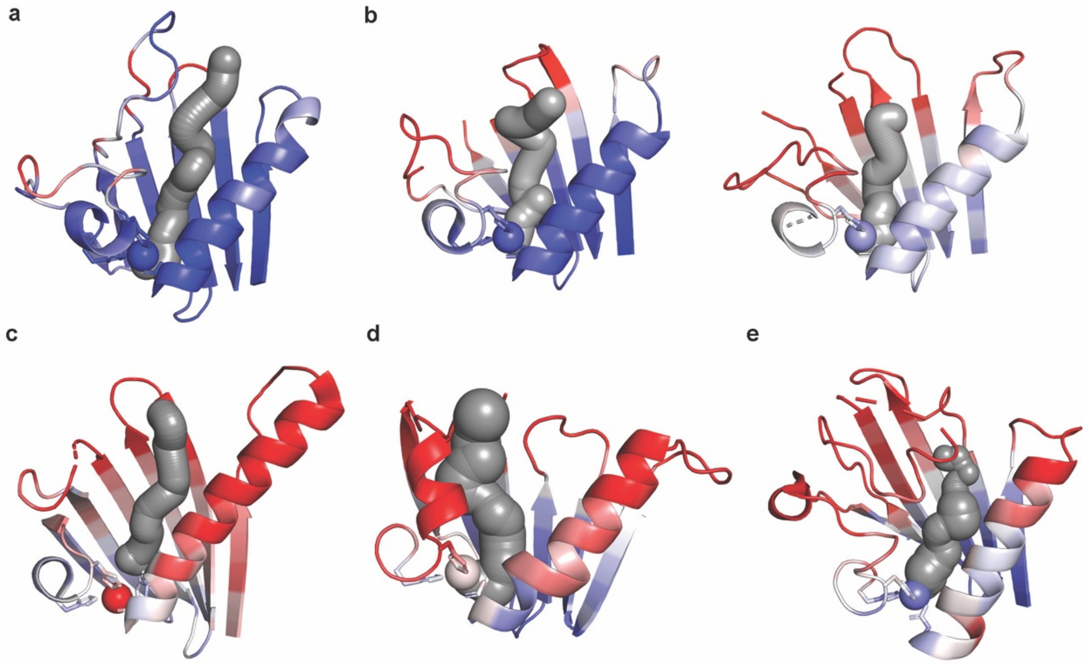

Supplementary Figure 19. A tunnel that spans a similar region to that observed in GxtA was also observed in NdmA, NdmB, KshA, CntA, and DdmC. (a) The structure of GxtA with its calculated tunnel determined in this work is provided for comparison. (b) Both NdmA (left) and NdmB (right) have tunnels that lead from the surface of the protein to the non-heme iron center (PDB: 6ICK, 6ICL)[3]. (c) A tunnel can also be calculated in KshA that is oriented similarly to that observed in GxtA (PDB: 4QCK)[4]. (d) The CntA (PDB: 6Y9D)[5] tunnel occludes visualization of the disordered loop that is shown in Supplementary Figure 15d. (e) DdmC (PDB: 3GKE)[6] also showcases a tunnel for substrate access that appears to be gated by the flexible loop that connects β13 and β14.

Supplementary Table 1. Data collection and refinement statistics.

	β-STOH-SxtT 7SZH	β-STOH-GxtA 7SZF	STX-GxtA 7SZE	Xe-GxtA[§] 7SZG
**Data collection**				
Space group	*C*222	*P*12$_1$1	*P*12$_1$1	*P*12$_1$1
Cell dimensions				
a, *b*, *c* (Å)	153.3, 158.5, 115.8	74.73, 96.93, 80.81	74.98, 96.93, 80.67	75.20, 96.64, 80.72
α,β,γ (°)	90, 90, 90	90, 107.0, 90	90, 106.91, 90	90, 107.1, 90
Resolution (Å)	50-1.79	50-1.79	50-1.74	50-2.69
R_{meas}[‡]	0.068 (1.702)	0.062 (0.723)	0.076 (1.035)	0.141 (1.393)
$I/\sigma I$[‡]	22.05 (1.86)	14.77 (1.93)	16.75 (2.05)	19.63 (2.42)
Completeness (%)[‡]	98.5 (97.8)	98.2 (96.8)	96.9 (96.1)	99.7 (100)
Redundancy[‡]	13.7 (13.4)	3.5 (3.3)	7.0 (6.8)	18.5 (17.5)
CC1/2[‡]	1.000 (0.815)	0.999 (0.737)	0.999 (0.756)	0.999 (0.793)
**Refinement**				
Resolution (Å)	1.79	1.79	1.74	2.69
No. reflections	129304	102236	109917	30537
R_{work} / R_{free}	0.170, 0.198	0.158, 0.197	0.161, 0.197	0.199, 0.235
No. atoms				
Protein	8073	7900	8165	7721
[2Fe-2S]	12	12	12	12
Non-heme Fe	3	3	3	3
Glycerol	108	84	72	36
Sulfate	30	-	-	-
Water	834	926	939	254
STX molecule	60	60	63	-
Cl	2	7	8	3
Xe	-	-	-	3
DTT	-	-	-	32
B-factors				
Overall	44.57	31.34	31.40	50.62
Protein	43.27	30.22	30.12	50.53
STX molecule	51.40	30.59	38.16	-
[2Fe-2S]	30.34	22.14	20.86	40.48
Non-heme Fe	29.75	20.87	20.51	46.13
Glycerol	65.40	49.63	53.31	66.05
Sulfate	90.54	-	-	-
Water	51.74	39.33	40.45	61.77
Cl	76.98	40.26	47.40	61.47
Xe	-	-	-	82.60
DTT	-	-	-	64.47
R.m.s. deviations				
Bond lengths (Å)	0.007	0.007	0.008	0.011
Bond angles (°)	0.918	0.897	0.950	1.586

[‡]Values in parentheses are for highest-resolution shell.
[§]Bijvoet pairs were not merged during data processing

Supplementary Table 2. Refined structure geometry statistics.

Structure	Missing residues	Ramachandran favored	Ramachandran allowed	Ramachandran disallowed	Rotamer outliers
β-STOH-SxtT	A: 1-2, 207-208, 297-303; B:297-303; C:203-211, 298-303	96.96%	3.04%	0%	0.99%
β-STOH-GxtA	A: 1,204-211, 297-303; B: 1, 203-210, 298-304; C: 1, 297-303	97.14%	2.86%	0%	0.33%
STX-GxtA	A: 201-211, 297-303; B: 300-304; C: 297-304	96.98%	3.02%	0	0.33%
Xe-GxtA	A: 1-2, 203-212, 297-303; B: 1, 203-211, 297-304; C: 298-304	96.71%	3.29%	0	0.92%

Supplementary Table 3. Sequences of primers used in this study.

Primer name	Primer DNA sequence
SxtT_M255Y	5' – gatgaagataatagcgttctgcgttatctgattatgtggaatggtagcga – 3'
SxtT_T276V	5' – aaatgctgaccgaatatgatgaagtcatcgaacaggatattcgcattc – 3'
SxtT_W214M	5' – agccatattgaagatgatagcatggtgaattggtttcgtctgag – 3'
SxtT_S230T	5' – cgctgtgtcagtattgtgttaccgaaagtccgg – 3'
SxtT_S232A	5' – gctgtgtcagtattgtgttagcgaagctccggaaatgcgta – 3'
SxtT_I237T	5' – gaaagtccggaaatgcgtactgttgatctgatgacc – 3'
SxtT_R204K	5' – tgggcaaatatcagtttcagaccagcaagattgttagccatattgaagatgatag – 3'
SxtT_F200V/Q201H	5' – gaccatgggcaaatatcaggttcataccagcaagattgttagc – 3'
SxtT_I205F/V206N/S207N	5' – gcaaatatcaggttcataccagcaagtttaataaccatattgaagatgatagctgggtga – 3'
SxtT_H208S/I209T/E210K	5' – aggttcataccagcaagtttaataacagtactaaagatgatagctgggtgaattggtttc – 3'

Supplementary References

1 Knapp, M., Mendoza, J. & Bridwell-Rabb, J. in *Encyclopedia of Biological Chemistry, 3rd Edition* (2021).

2 Eisenberg, D., Schwarz, E., Komaromy, M. & Wall, R. Analysis of membrane and surface protein sequences with the hydrophobic moment plot. *J Mol Biol* **179**, 125-142, doi:10.1016/0022-2836(84)90309-7 (1984).

3 Kim, J. H. *et al.* Structural and Mechanistic Insights into Caffeine Degradation by the Bacterial N-Demethylase Complex. *J Mol Biol* **431**, 3647-3661, doi:10.1016/j.jmb.2019.08.004 (2019).

4 Capyk, J. K., D'Angelo, I., Strynadka, N. C. & Eltis, L. D. Characterization of 3-ketosteroid 9a-hydroxylase, a Rieske oxygenase in the cholesterol degradation pathway of *Mycobacterium tuberculosis*. *J. Biol. Chem.* **284**, 9937-9946 (2009).

5 Quareshy, M. *et al.* Structural basis of carnitine monooxygenase CntA substrate specificity, inhibition and inter-subunit electron transfer. *J Biol Chem*, doi:10.1074/jbc.RA120.016019 (2020).

6 Dumitru, R., Jiang, W. Z., Weeks, D. P. & Wilson, M. A. Crystal structure of dicamba monooxygenase: a Rieske nonheme oxygenase that catalyzes oxidative demethylation. *J Mol Biol* **392**, 498-510, doi:10.1016/j.jmb.2009.07.021 (2009).

7 Daughtry, K. D. *et al.* Quaternary ammonium oxidative demethylation: X-ray crystallographic, resonance Raman, and UV-visible spectroscopic analysis of a Rieske-type demethylase. *J Am Chem Soc* **134**, 2823-2834, doi:10.1021/ja2111898 (2012).

8 Nojiri, H. *et al.* Structure of the terminal oxygenase component of angular dioxygenase, carbazole 1,9a-dioxygenase. *J Mol Biol* **351**, 355-370, doi:10.1016/j.jmb.2005.05.059 (2005).

9 Ashikawa, Y. *et al.* Electron transfer complex formation between oxygenase and ferredoxin components in Rieske nonheme iron oxygenase system. *Structure* **14**, 1779-1789 (2006).

10 Martins, B. M., Svetlitchnaia, T. & Dobbek, H. 2-Oxoquinoline 8-monooxygenase oxygenase component: active site modulation by Rieske-[2Fe-2S] center oxidation/reduction. *Structure* **13**, 817-824, doi:10.1016/j.str.2005.03.008 (2005).
